# Intra-Household and Close-Contact SARS-CoV-2 Transmission Among Children – a Systematic Review

**DOI:** 10.3389/fped.2021.613292

**Published:** 2021-04-09

**Authors:** Benedikt D. Spielberger, Tessa Goerne, Anne Geweniger, Philipp Henneke, Roland Elling

**Affiliations:** ^1^Center for Pediatrics and Adolescent Medicine, Medical Center-University of Freiburg, Freiburg, Germany; ^2^Institute for Immunodeficiency, Center for Chronic Immunodeficiency, Medical Center – University of Freiburg, Freiburg, Germany

**Keywords:** SARS-CoV-2, COVID-19, secondary attack rate, transmission, household, SAR, child

## Abstract

**Introduction:** The outbreak of the novel coronavirus disease (COVID-19) caused by the severe acute respiratory syndrome coronavirus 2 (SARS-CoV-2) has led to a range of emergency measures worldwide. Early in the pandemic, children were suspected to act as drivers of the COVID-19 spread in the population, which was based on experiences with influenza virus and other respiratory pathogens. Consequently, closures of schools and kindergartens were implemented in many countries around the world, alongside with other non-pharmaceutical interventions for transmission control. Given the grave and multifaceted consequences of contact restriction measures for children, it is crucial to better understand the effect size of these incisive actions for the COVID-19 pandemic. Therefore, we systematically review the current evidence on transmission of SARS-CoV-2 to and by children.

**Data Sources:** PubMed and preprints uploaded on medRxiv.

**Study Selection:** Original research articles, case reports, brief communications, and commentaries were included into the analysis. Each title or abstract was independently reviewed to identify relevant articles. Studies in other languages than English were not included.

**Data Extraction:** Two reviewers independently reviewed the selected studies. Extracted data included citation of each study, type of healthcare setting, location of the study, characteristics of patient population, and reported outcomes.

**Results:** Data on transmission of SARS-CoV-2 on or by children is scarce. Several studies show a lower seropositivity of children compared to adults, suggesting a lower susceptibility of especially younger children. Most insight currently comes from household studies suggesting, that children are predominantly infected by their household contacts. The contagiousness however, seems to be comparable between children and adults, based on our meta-analysis of included studies.

**Conclusions:** Larger and systematic studies are urgently needed to better understand the age dependent patterns of SARS-CoV-2 transmission and thereby design more effective non-pharmaceutical interventions to reduce disease transmission.

## Introduction

### Rationale

Coronaviruses are a large family of single-stranded RNA viruses, four of which commonly circulate among humans (229E, HKU1, NL63, OC43) (1). Infections with these common coronaviruses (cCoV) typically cause respiratory or gastrointestinal symptoms with a usually mild to moderate course of disease. Overall, in 4–6% of children hospitalized for respiratory symptoms, cCoV can be isolated (2–4). However, two novel CoVs, SARS-CoV and MERS-CoV, which have emerged in the last decade, are associated with severe illness and death (5, 6).

Most recently, in December 2019, an uncommon series of severe pneumonia in the city of Wuhan, China led to the identification of a novel coronavirus, initially termed 2019-nCoV. The virus was renamed to SARS-CoV-2, when it became clear that it was genetically related to SARS-CoV (7). The disease caused by SARS-CoV-2 was named COVID-19 by the WHO on February 11th 2020^1^.

Within 12 months after the first identified cases, the COVID-19 pandemic has risen to more than 85 million cases worldwide, and has claimed close to two million lives. COVID-19 is reported to have a mild course in about 80%, and a severe to critical course in about 20% of infected adults. It predominantly causes fever, cough, and severe pneumonia including acute respiratory distress syndrome (ARDS), but other manifestations with predominantly gastrointestinal or neurologic symptoms have been reported (8–12).

Most children infected with SARS-CoV-2 have only mild symptoms like fever, cough, or gastrointestinal symptoms, the latter of which occur more often than in adults (13–15). In addition, since early May 2020, several countries have reported on a severe hyperinflammatory syndrome in children associated with SARS-CoV-2 infection showing some overlap with Kawasaki disease, hemphagocytic lymphohistiocytosis, and macrophage activation syndrome. The condition is denominated pediatric inflammatory multisystem syndrome temporally associated with SARS-CoV-2 (PIMS-TS) in the UK, or multisystem inflammatory syndrome in children (MIS-C) in the USA (16–19).

In order to reduce transmission and control the spread of the virus, strict travel restrictions and different degrees of social distancing measures have been implemented in many countries, starting early in 2020. Closures of schools and kindergartens are accepted as effective measures to limit influenza virus outbreaks, since school-based transmission is a recognized driver of the disease spread (20, 21). Although it was not clear whether school measures are comparably effective in coronavirus outbreaks and evidence from previous coronavirus outbreaks suggested a low transmission risk in schools (22), many countries implemented large or national school closures in March 2020 (23). Moreover, contact restriction measures have increased the contact time between children and other household members. Accordingly, more than 6 months after the WHO has declared COVID-19 a pandemic, the contribution of children, and in particular the impact of school closures and stay-at-home policies on the dynamics of the pandemic remains unclear.

In order to make progress in this area, we have systematically review the available evidence on the role of children as drivers of the pandemic, and of school and kindergarten closures as means to limit SARS-CoV-2 spread in the community.

### Objectives

The aim of this systematic review was to analyze and review the evidence as of August 11th 2020 on intra-household and close-contact transmission dynamics of SARS-CoV-2 among children with a special focus on the susceptibility and contagiousness of children and adolescents. We addressed the following key questions:

- What is the susceptibility to a SARS-CoV-2 infection of children compared to adults?
- To what extent do children and adolescents spread SARS-CoV-2 in a household or close-contact setting compared to adults?
- Have differences between different age groups like toddlers, teens, and adolescents been observed regarding virus transmission?

## Methods

### Protocol and Registration

We conducted a search on PubMed and on medRxiv on August 11th 2020 evaluating all studies for inclusion that were presenting data on SARS-CoV-2 transmission on or by children and adolescents. Infection or transmission had to be confirmed by SARS-CoV-2 PCR or serology. All studies, irrespective of number of participants, interventions or timing were evaluated. Where possible, raw data on index cases, secondary cases, and routes of infection were extracted and quantitatively analyzed.

### Eligibility Criteria

Our inclusion criteria were as follows:

1. Data source: published, peer reviewed, or preprint, i.e., not peer reviewed articles.
2. Publication type: observational studies (cross-sectional, case-control, retrospective, prospective, mixed-cohort designs), intervention studies, guidelines, commentaries, conference abstracts. Only articles written in English were included.
3. SARS-CoV-2 infection proven by serology or by RT-PCR.
4. Specific reporting on SARS-CoV-2 transmission from children, or on children in households, communities, schools, or kindergartens since the onset of the SARS-CoV-2 pandemic. We did not formally define age thresholds for children and adults, but used the given age ranges in order to not miss out any studies.

- Contact tracing studies: Report on either secondary infections in children and adolescents after contact with an adult index patient, or report on secondary infections of adults or children and young adults with a pediatric index patient.
- Population seroprevalence studies had to provide information on SARS-CoV-2 sero-prevalence in children and adults separately.

Exclusion criteria were as follows:

We excluded studies that were conducted in any other language than English, that reported only about vertical transmission from mother to child in a perinatal setting, and where ages were not clearly classified, i.e., adult and pediatric data could not be separately evaluated.

Information sources: PubMed, medRxiv.

**Figure 1:**
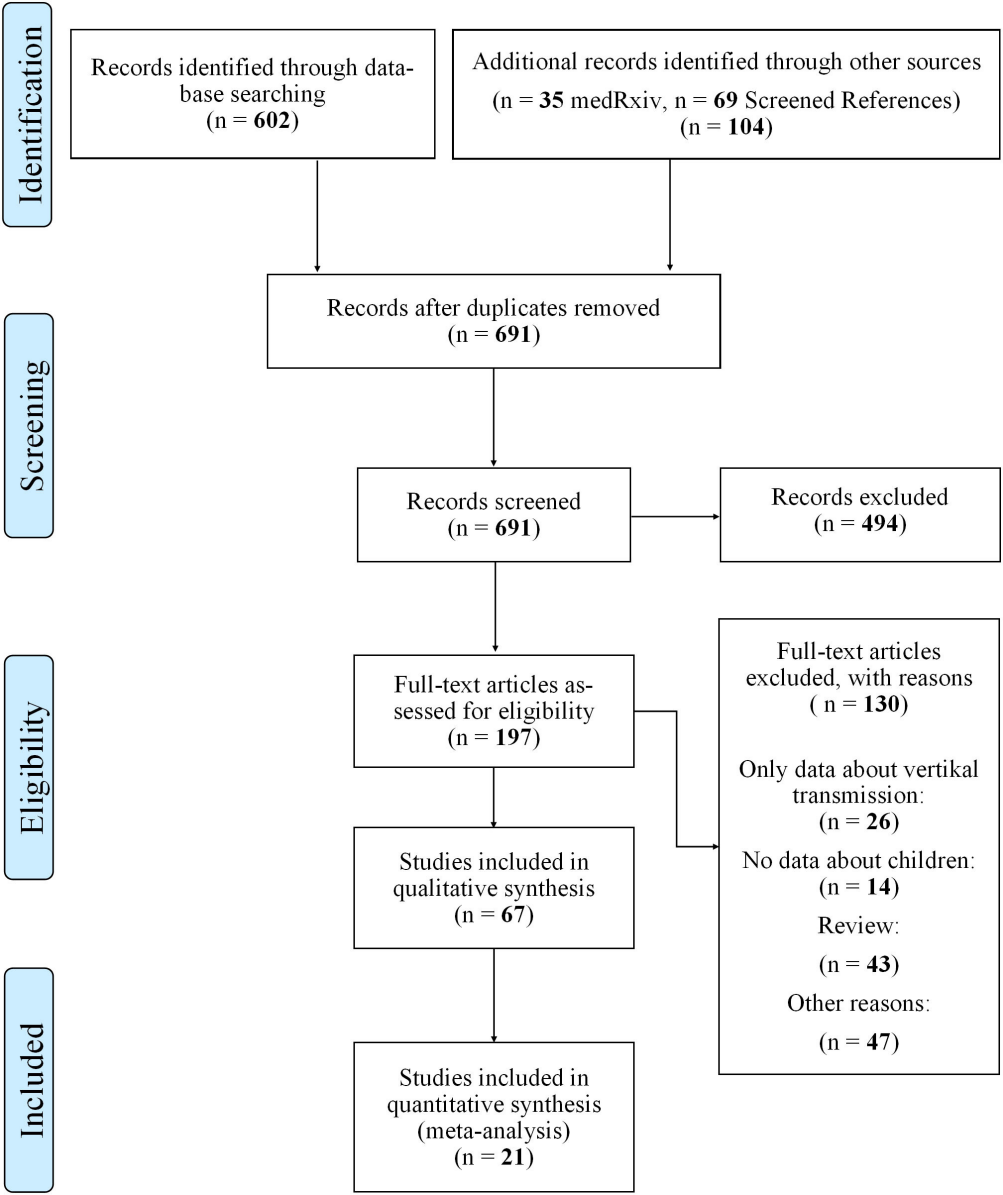
PRISMA flow diagram for search.

Search: Figure 1 describes our flow diagram of study selections, conducted on August 11th 2020.

The search on PubMed was conducted with the following search string: (COVID or COVID-19 or COVID-19 or SARS-CoV-2 or nCov2019) AND (child or children or adolescent^*^) AND (transmission or household or community), restricted to studies in English, on humans and with abstracts.

The search in medRxiv was conducted with the following search terms: COVID^*^ and Child^*^ and transmission^*^, COVID^*^ and Child^*^ and household^*^, SARS^*^ and child^*^ and household^*^ SARS^*^ and Child^*^ and transmission^*^, SARS^*^ and adolescent^*^ and transmission^*^, SARS^*^ and adolescent^*^ and household^*^, COVID^*^ and adolescent^*^ and household^*^, COVID^*^ and adolescent^*^ and transmission^*^.

We searched cited references in potentially eligible studies for additional candidate studies. Additional studies were also identified by the authors and through their professional network.

Study selection: The abstracts and titles of retrieved studies were screened to identify eligible studies by one researcher (BDS). The full text of potentially eligible studies was then retrieved and independently reviewed in duplicate for eligibility based on the inclusion and exclusion criteria by BDS, TG, and RE.

Data collection process: Data were collected in a predefined format by one reviewer, and reviewed by a second scientist to reduce missed studies. Results were compared, and disagreements resolved by discussion.

Data items: The following types of data were extracted from each study: publication status (preprint, peer reviewed), characteristics (e.g., study type, region, time span), participant characteristics (e.g., age, age range, gender), tests performed (including laboratory tests, serology, PCR and cut-off-values if available), sociodemographic factors (number of adults or children per household, number of rooms, garden, etc.). If available, data on shutdown procedures, school or kindergarten closures etc. were included. All eligible articles were subject to meta-analysis.

### Figures and Data Acquisition for the Figures

**Figure 2:**
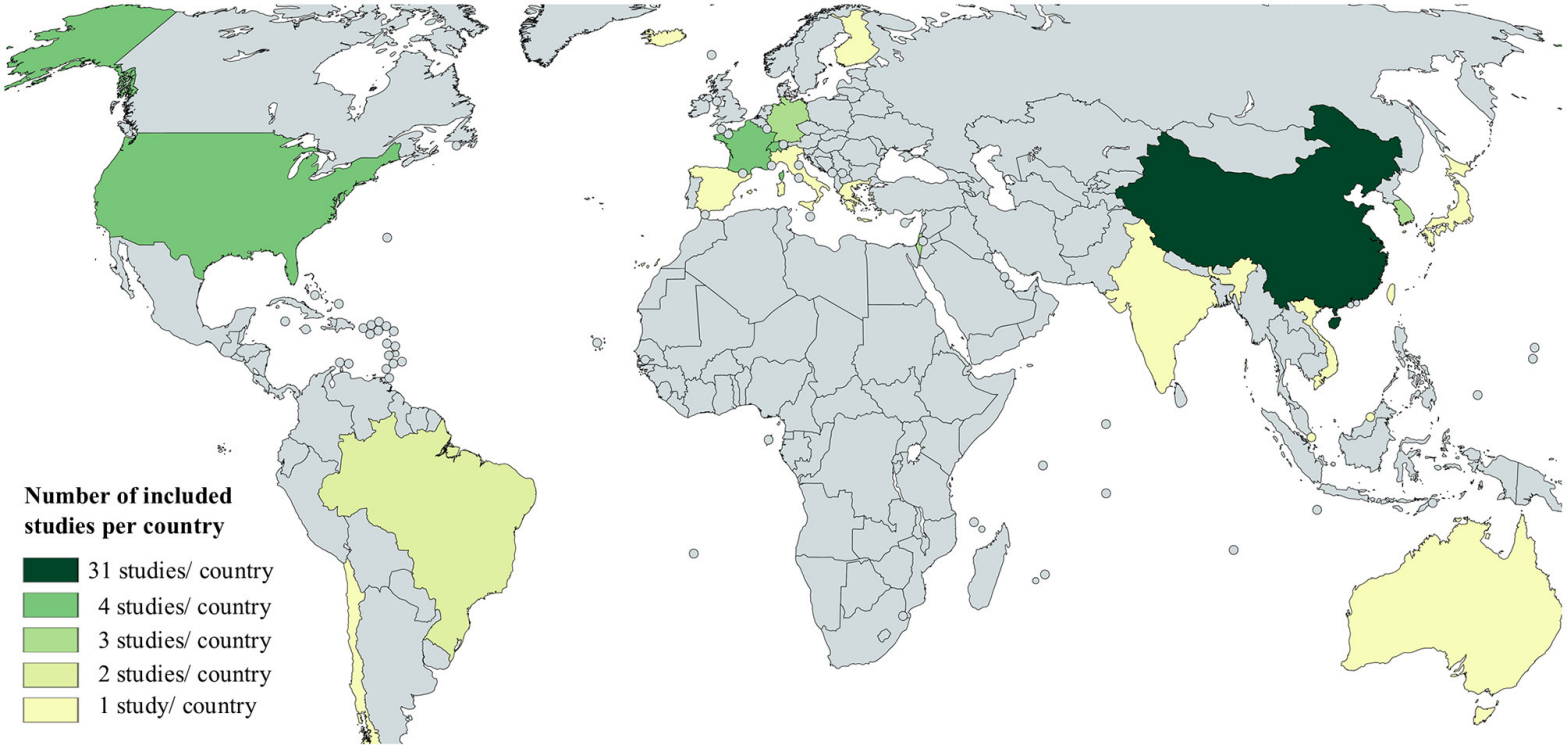
Overview of the origin of studies included in this review.

**Figure 3:**
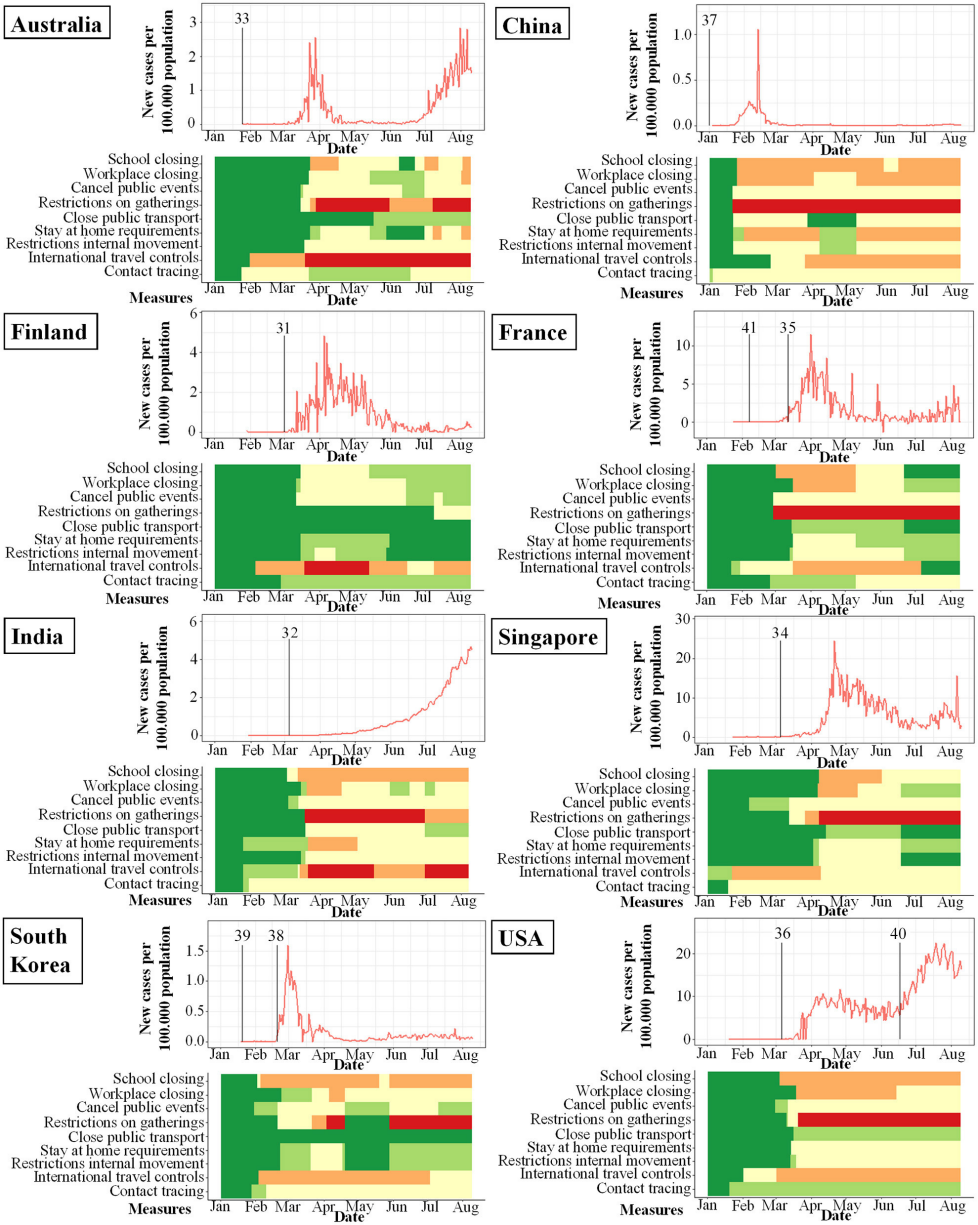
Studies included in meta-analysis and their study start (indicated by vertical bar) in proportion to incidence of new infections with SARS-CoV-2 per 100.000 per day (y-axis) and changes over time (x-axis). Local response reactions to reduce spread of SARS-CoV-2 are shown below the x-axis in colored bars. Green bars mean no restrictions, red fullrestrictions, e.g., shut down of transport, no gatherings. Dataset of the figure is available in the Supplementary Material.

**Figure 4:**
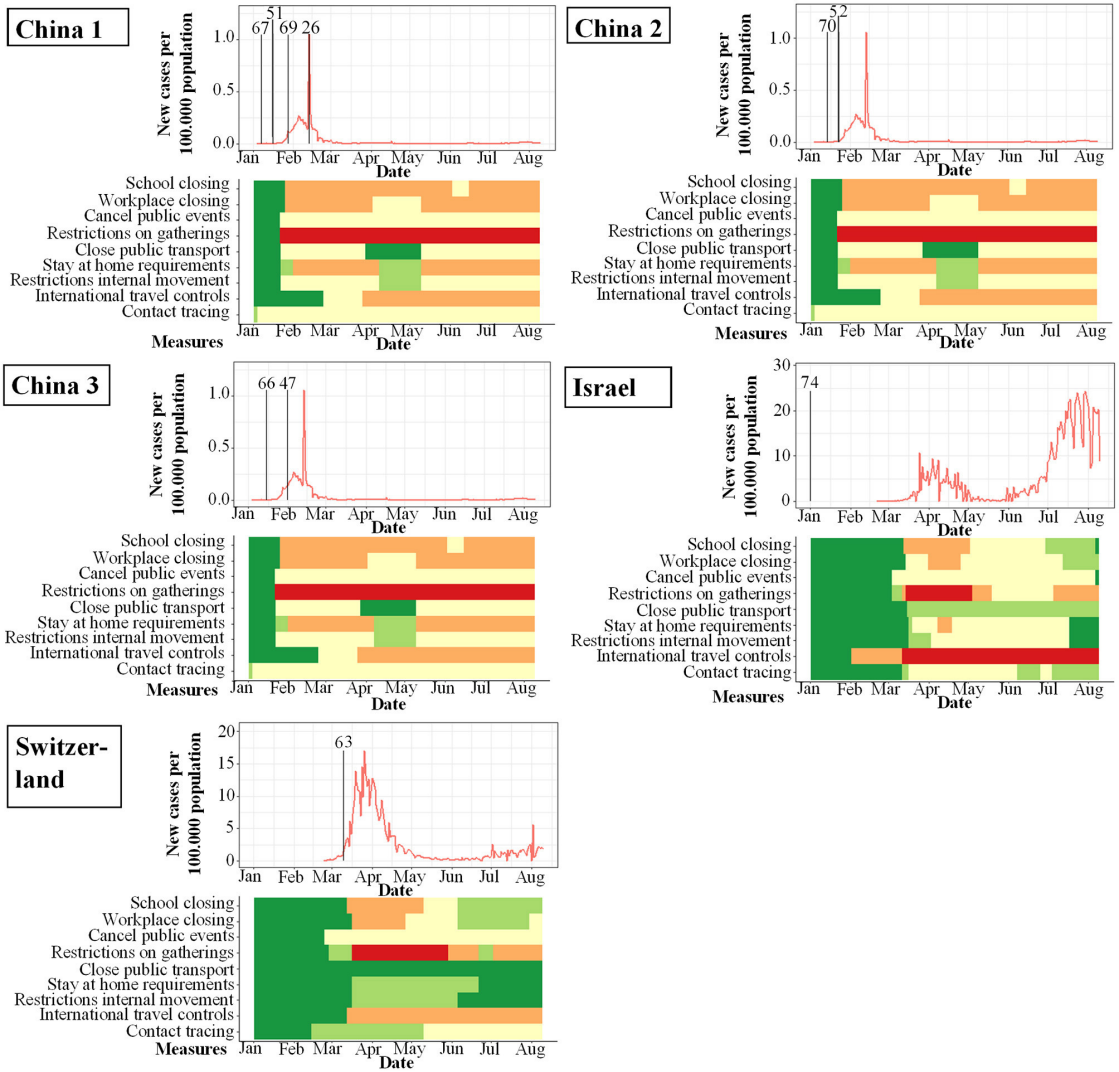
Studies included in qualitative analysis and their study start (indicated by vertical bar) in proportion to incidence of new infections with SARS-CoV-2 per 100.000 per day (y-axis) and changes over time (x-axis). Local response reactions to reduce spread of SARS-CoV-2 are shown below the x-axis in colored bars. Green bars mean no restrictions, red full restrictions, e.g., shut down of transport, no gatherings. Dataset of the figure is available in the Supplementary Material.

**Figure 5:**
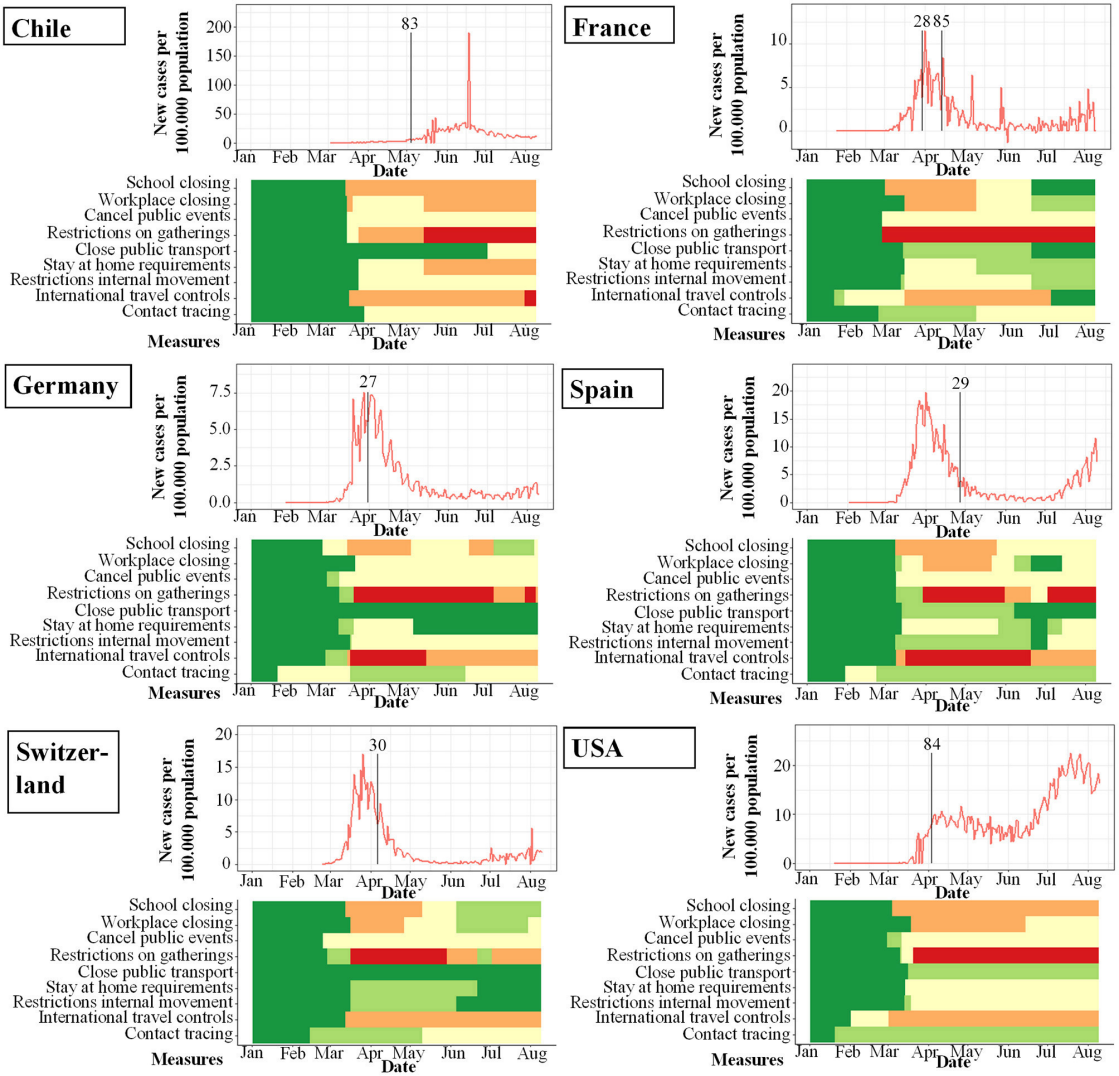
Seroprevalence studies and their start (indicated by vertical bar) in proportion to incidence of new infections with SARS-CoV-2 per 100.000 per day (y-axis) and changes over time (x-axis). Local response reactions to reduce spread of SARS-CoV-2 are shown below the x-axis in colored bars. Green bars mean no restrictions, red fullrestrictions, e.g. shut down of transport, no gatherings. Dataset of the figure is available in the Supplementary Material.

**Figure 6:**
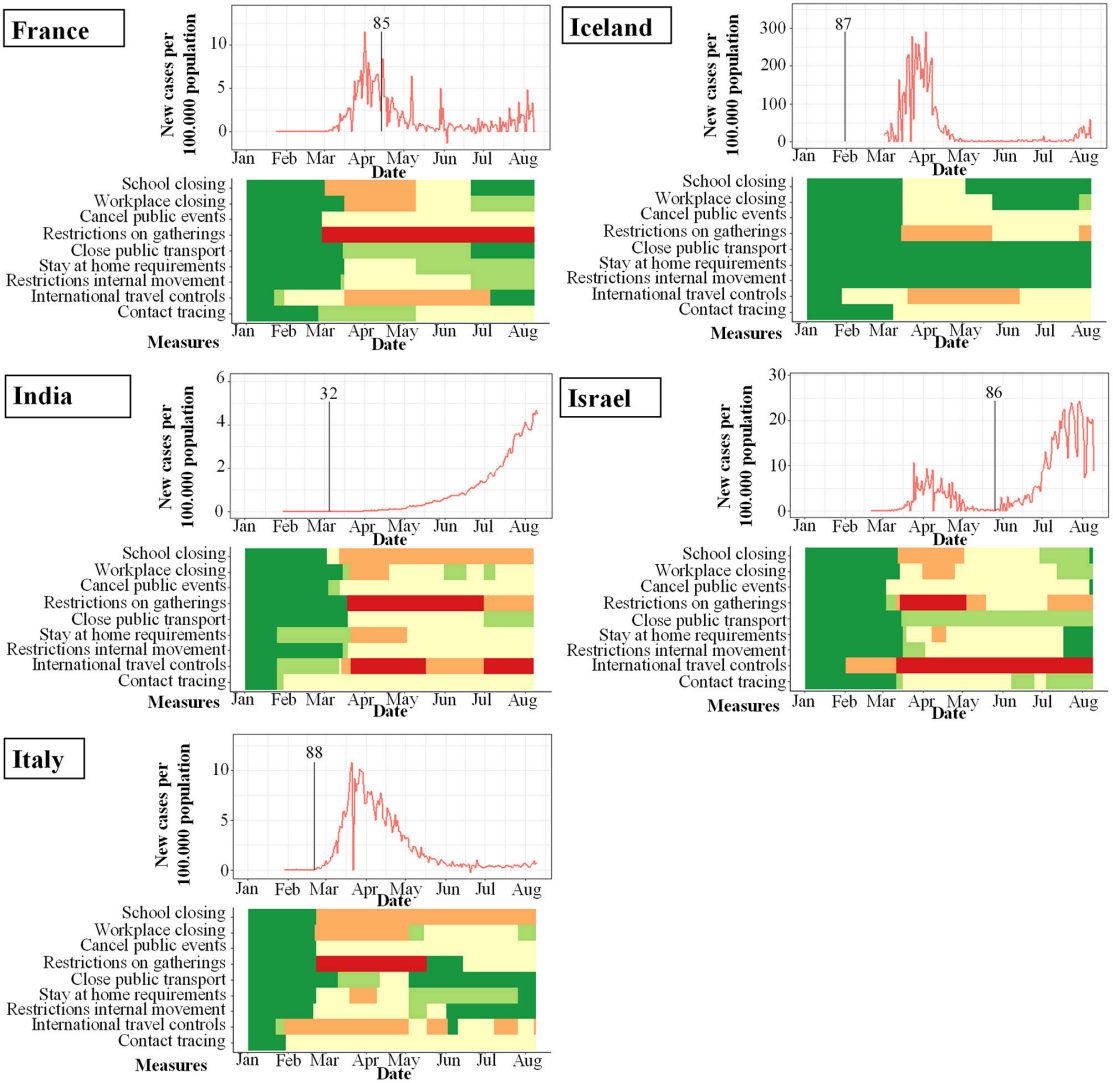
PCR-prevalence studies and their start (indicated by vertical bar) in proportion to incidence of new infections with SARS-CoV-2 per 100.000 per day (y-axis) and changes over time (x-axis). Local response reactions to reduce spread of SARS-CoV-2 are shown below the x-axis in colored bars. Green bars mean no restrictions, red fullrestrictions, e.g., shut down of transport, no gatherings. Dataset of the figure is available in the Supplementary Material.

Figure 2 was created with the free online tool MapChart (Mapchart.net). The relative number of newly confirmed cases per 100.000 population in Figures 3–6 as calculated using the current estimates of the UN Department of Economics and Social Affairs, Population Dynamics (https://population.un.org/wpp/; Accessed 20.08.2020 15:00) and the WHO Coronavirus Disease (COVID-19) Dashboard (https://covid19.who.int/table; Accessed 20.08.2020 15:00). For easier comparison, we displayed data of the Government Response to Corona as published by the Oxford COVID-19 Government Response Tracker (24). The corresponding Codebook is available under https://github.com/OxCGRT/covid-policy-tracker/blob/master/documentation/codebook.md, accessed 20.08.2020 15:00. The R software (version 3.5.2) was used for visualization including the ggplot2 (version 3.1.0) and ggpubr (version 0.2) package. The written code is included in the supplements.

### Meta-Analysis

Secondary attack rates (SAR) and the corresponding standard error were calculated using extracted data for numbers of secondary cases and number of susceptible contacts from the studies included in the review. Calculations of SAR were performed for both adult and child index patients, depending on the availability of data from the respective studies. A random effects model using a restricted maximum likelihood estimator model was chosen for meta-analysis, giving a point estimate and 95% confidence interval (CI) for SAR by index patient. I-squared is reported as inconsistency index, indicating how much variation in the pooled estimates is due to heterogeneity. Tau-squared is reported as heterogeneity-parameter, representing between-study variability. Based on recommendations by McCandless and Oliva (25), studies with number of susceptible contacts <20 or number of susceptible contacts minus secondary cases <5 were excluded from meta-analysis to minimize statistical heterogeneity. Meta-analysis was conducted in Stata statistical software (Release 14) using the metan-command.

## Results

### Study Selection

As shown in Figure 1, we identified 602 articles in PubMed, 35 additional articles in MedRxiv and further 69 after screening the references. After duplicates were excluded, 691 articles were screened for eligibility by reading abstracts and titles and 494 were excluded. Of the remaining 197 articles we excluded a total of 130 after reading the fulltexts and remained with 67 articles appropriate for qualitative analysis.

Twenty-one articles contained detailed data on secondary attack rate (SAR) in children and epidemiologic data, so that those were subject to further quantitative analysis.

### Study Characteristics

As illustrated in Figure 2, the majority of studies included in this review were conducted in China (*n* = 31), followed by four studies each from France, Switzerland, and the United States of America. Three studies from Germany, Israel, and South Korea and two studies from Brazil were included in this review. One study each was included from Brunei Darussalam, Chile, Spain, Italy, Greece, Iceland, Finland, India, Japan, Singapore, Taiwan, Australia, and Vietnam.

While the amount of studies included reflects a worldwide spectrum, the period when the studies were conducted in relation to the incidence of documented SARS-CoV-2 infections was comparably homogenous as shown in Figures 3–6. The majority of studies, especially in China, was conducted when the incidence of confirmed SARS-CoV-2 infections was very low. Only the studies of Wang (26), Streeck (27), Fontanet (28), Pollán (29), and Stringhini (30) were performed when the incidence in the respective countries was high. When taking national reactions against spreading of the pandemic into account, we found that most of the studies were at least initiated before travel restrictions, stay-at-home policies, school closures, and restrictions on gatherings were established. This suggests that the results were not excessively influenced by those measures.

### Results of Individual Studies

#### Contact Tracing Studies Suitable for Meta-Analysis

We identified and analyzed 11 contact tracing studies. A majority of studies were performed in the early phase of the pandemic in China. Further reports are from Finland, India, Australia, Singapore, and the USA.

#### Contact Tracing Studies With Adult or Pediatric COVID-19 Index Patient

Dub et al. (31) from Finland performed a retrospective study after two school outbreaks of SARS-CoV-2 with one infected adult and one infected child respectively. For the adult index case, neutralizing IgG-antibodies for SARS-CoV-2 were identified in 17% of exposed students (7 of 42) and 11% of exposed adults (1 of 9). In the child index case, 87 exposed children underwent serology and 82 children nasal swab and RT-PCR. All children were found to be negative in both tests, indicating that no transmission of SARS-CoV-2 has occurred by this mildly symptomatic child.

In one of the largest PCR-based studies on SARS-CoV-2 prevalence to date, Laxminarayan et al. (32) analyzed the disease surveillance data collected through June 4th 2020 in the provinces Tamil Nadu and Andhra Pradesh in India. These comprised an impressive number of 33.584 RT-PCR confirmed COVID-19 cases. SAR estimates ranged from 1.0% (0.0–5.4%) in healthcare settings to 2.6% (1.6–3.9%) in the community and 9.0% (7.5–10.5%) in the household; in total, 48.3% of all positive contacts were traced to an index case in the household. The SAR for adult index cases in a household-setting was 9.2% (95%CI 8.2%; 10.2), while the SAR for child index patients in a household setting showed comparable levels of 7.83% (95%CI 3.7–11.9), suggesting a comparable contagiousness with the limitation that the 95% confidence interval was greater in children.

Macartney et al. (33) prospectively investigated all COVID-19 cases in children and adults that attended a school or early childhood education and care (ECEC) setting in New South Wales, Australia from January to April 2020. In a total of 25 schools and ECEC facilities, 12 children and 15 adults were identified as COVID-19 index cases. 633 of 1,448 (43.7%) contacts were evaluated by RT-PCR, serology or both, and 18 secondary cases were identified (SAR 1.2%). Four adult index cases in secondary schools did not lead to secondary infections in 39 exposed adults and 87 exposed children (total 126 contacts). In primary schools, 4 infected adults had a total of 173 contacts, with a SAR of 2/37 (5.4%) in adults and 1/136 (0.7%) in children. In ECEC settings, 7 adult index patients had 412 contacts, with a SAR of 32.4% in adults and 6.5% in children. In ECEC settings, 3 children had 37 adult and 85 child contacts, and none of them was tested positive by RT-PCR or serology. In summary, this study did not find secondary cases of infectious children in ECEC or school settings, while infectious adults in the same environment led to varying SARs between 1.7 and 32.4%.

Yung et al. (34) retrospectively assessed SARS-CoV-2 transmission in 137 households in Singapore with one adult index case each. Thirteen of 213 exposed children under 16 years were infected, resulting in a SAR of 6.1% for children. In an age-stratified analysis, the attack rate was 1.3% among children under the age of 5 years, 8.1% among those of 5–9 years, and 9.8% among those of 10–16 years of age.

A French study investigated potential transmission patterns in three households living closely together in a rural area (35). Three adult index cases were in contact with in total 11 children and 16 adults. No children, but 6 adults were positive for antibodies against SARS-CoV-2, resulting in a SAR of 37.5% in adults, and 0% in children.

The morbitidy and mortality report by James et al. (36) highlights virus transmission by two adults involved in bible courses and services at a church in Arkansas, USA. A total of 92 persons were in close contact while e.g., singing in church. Strikingly, 33 of 60 adults, but only 2 of 32 children tested positive for SARS-CoV-2, resulting in a SAR of 55 and 6.25% respectively. When stratified by age, attack rates were significantly lower in people ≤18 years (6.3–25.0%) than in adults between 19 and 64 years (59.4–82.6%) (*p* < 0.01). The relative risk ratios for people ≤18 years was 0.1–0.3 as compared to adults.

Li et al. (37) analyzed data from two hospitals comprising 105 adult patients with a positive SARS-CoV-2 RT-PCR and 392 household contacts. Secondary virus transmission occurred in 64 of 392 household contacts (16.3%). The SAR in children was 4% as compared to 17.1% for adults. Interestingly, self-quarantine of the index patient after onset of symptoms was very effective as compared to no isolation, illustrated by a large difference in SAR (0 vs. 16.9%).

Park et al. retrospectively studied 5,553 adult and 153 children index cases infected with SARS-CoV-2 in South Korea (38). Stratified by age, the SAR was lowest in index patients aged 0–9 years (5.3% (95%CI 1.3–13.7) and highest in those at 10–19 years (18.6%, 95%CI 14.0–24.0), compared to a SAR of 11.6% among 5,553 adult index patients.

#### Contact Tracing Studies With Pediatric COVID-19 Index Patient

We included seven studies, where a child was the most likely COVID-19 index patient, into the analysis. In general, the mild or even absent symptoms of infected children significantly impede the generation of unequivocal transmission chains in contact tracing studies. Nevertheless, the following studies described scenarios, where it was plausible to define a child as the most likely index case.

Kim et al. (39) retrospectively analyzed all pediatric COVID-19 index cases and household members reported in South Korea from January 20th till April 6th 2020. Of a total of 107 pediatric SARS-CoV-2 infections and 248 tracked household contacts, only one secondary household transmission was identified. The authors followed 4.3 (range 1–67) household contacts on average per pediatric SARS-CoV-2 index for a median of 14 days by RT-PCR; serology tests were not performed. This resulted in a household SAR of 0.5% (95% CI 0.0–2.6), when a child was the index patient. Transmission was confirmed from a 16 years old girl to her 14 years old sister, while both parents remained negative on repeated RT-PCR tests.

A very different transmission pattern was found by Szablewski et al. (40), analyzing the outbreak of SARS-CoV-2 in a camp in Georgia, USA. Despite all attendees and staff had a negative RT-PCR a minimum of 12 days before attending the camp, one teenage member of staff developed chills and subsequently was tested positive for SARS-CoV-2 by RT-PCR. 260 of 344 (SAR 75.6%) tested positive on RT-PCR in the 14 days after the camp was shut down, Overall, the SAR was at least 44% (260 of 597 attendees and staff) and varied between 51% among those aged 6–10 years, 44% among those aged 11–17 years, and 33% among those aged 18–21 years. This report strongly suggests that adolescent children can very effectively spread COVID-19.

Danis et al. (41) report of a local outbreak in a French alpine ski chalet, where one adult index patient caused infection with SARS-CoV-2 in 11 of 15 individuals (SAR 73%). However, one pediatric case, with coinfection of SARS-CoV-2, a non-specified picornavirus and influenza A visited 3 different schools while being infective, resulting in 1 of 172 (0.58%) contacts testing positive for SARS-CoV-2.

#### Transmission Risk of SARS-CoV-2 by Children – Meta Analysis

**Figure 7:**
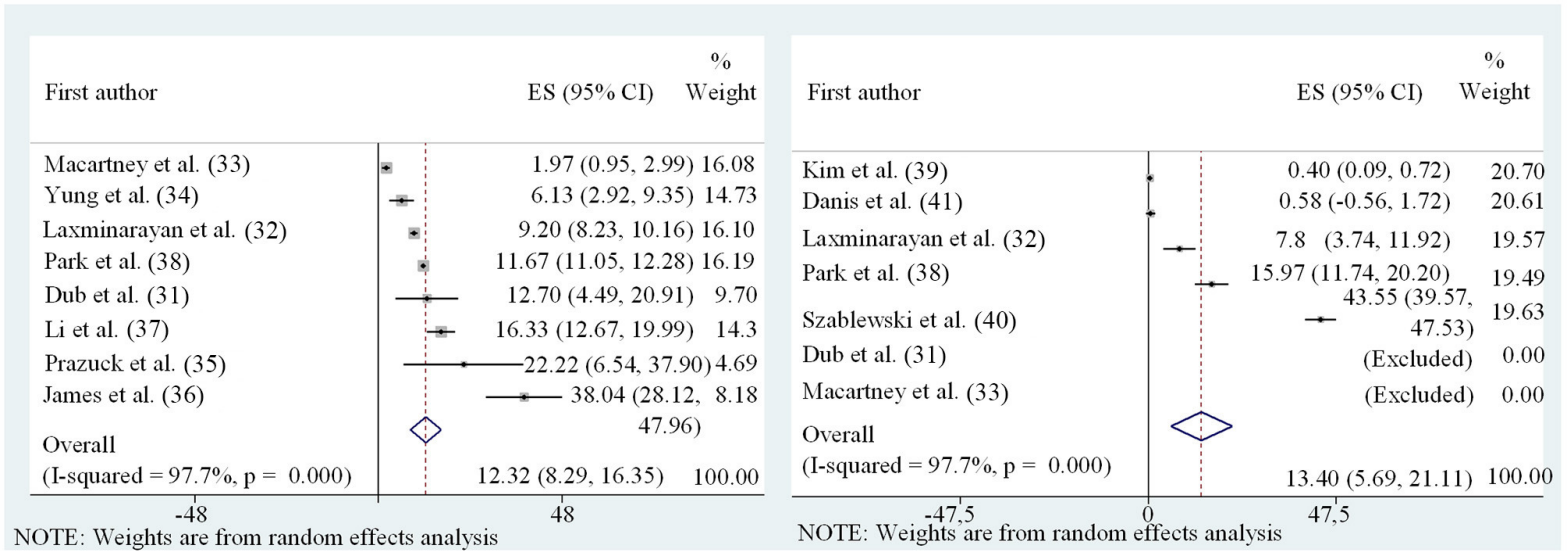
Forest plot of meta-analysis of secondary attack rates of child **(Left)** and adult **(Right)** index persons.

While there is considerable evidence that especially younger children are less susceptible to SARS-CoV-2 infections, data on the frequency of SARS-CoV-2 transmission by children (i.e., their contagiousness) are scarce. It should be noted that the two factors susceptibility and contagiousness are often intermixed, but are very distinct parameters that need to be used with great care. As an example, in a scenario of child index case not causing a high rate of secondary cases among other children in a daycare setting, it is inappropriate to conclude that children are less infectious. At the same time, this phenotype can be attributed to the lower susceptibility of SARS-CoV-2 infection after exposure, and the individual importance of these factors is unknown. The transmission risk of infected children vs. adults can only be estimated in settings where a definite and unique index patient simultaneously exposes a comparable cluster of adults and children e.g., in a household setting. However, these settings are difficult to define, given the often asymptomatic nature of SARS-CoV-2 infections in children, where previous intra-familial transmissions can rarely be excluded. Moreover, many other variables, such as variable social behaviors between adults and children further complicate these analyses. Nevertheless, we aimed to undertake a meta-analysis of appropriate studies with (a) detailed data, (b) accurate and reasonable case definitions, and (c) a relatively large number of contacts. Since the data was highly heterogeneous, a random-effects model was chosen. Data were separately evaluated for adult and child index persons (Figure 7, summarized in Table 1). The high heterogeneity with partly contradictive data is reflected by both the size of error-bars and a wide variation of SARs. After conducting the meta-analysis, the pooled SAR for a child index was 13.40% (95%CI 5.7–21.1) compared to 12.32% (95%CI 8.3–16.4) in adults. On the basis of limited data and high heterogeneity, the analysis did not reveal evidence for significant differences regarding the contagiousness of children and adults with SARS-CoV-2 infections. These data have to be interpreted with caution however—given the limitations discussed above.

**Table 1:** Characteristics of studies included in quantitative analysis and their main important findings and numbers.

**First author**	**Study status; type**	**Country; timing of study**	**Setting; n(children); n(adults)**	**Method**	**Case definition**	**Testing**	**Age range (years): child; adult**	SAR index adult^*^	SAR index child^*^
Dub et al. (31)	Preprint	Finland; Early March 2020—not stated	Two school exposure incidents in the Helsinki area; 131 pupils; 9 school staff members	Retrospective investigation using short questionnaires and testing of participants	Positive RT-PCR or positive microneutralization testing	RT-PCR testing on nasopharyngeal swabs in exposure A; microneutralization testing and fluorescent microsphere immunoassay on serum specimens in exposure A and B	Not stated	12.70 (4.49; 20.91)	N.a.
Laxminarayan et al. (32)	Preprint	India; 05.03.2020–04.06.2020	Clusters defined by contact-tracing in the states Tamil Nadu and Andhra Pradesh; Tamil Nadu 42506; 476429 tested; Andhra Pradesh 30076; 446912 tested	Retrospective analysis of the surveillance program including contact tracing data	Positive RT-PCR	Initially RT-PCR for symptomatic individuals with history of travel or contact of confirmed case; expanded to all symptomatic individuals and asymptomatic contacts of confirmed cases between 20.-28.03.2020	0–17; ≥ 18	9.20 (8.23; 10.16)	7.83 (3.74; 11.92)
Macartney et al. (33)	Published; Peer reviewed	Australia; 25.01.2020–01.05.2020	School and early childhood education and care clusters in New South Wales (NSW); COVID-19 cases 98; 2,936 Contacts 249; 39	Prospective investigation of index cases and their close contacts; index identification through all confirmed Cases in NSW	Positive RT-PCR; first confirmed case who attended the facility while infectious	RT-PCR testing on nasopharyngeal swabs; 35% child close contacts and 46% adult close contacts tested; indirect immunofluorescence assay on blood specimens	0–18; ≥ 19	1.97 (0.95; 2.99)	N.a.
Yung et al. (34)	Online Report	Singapore; 05.03.2020–30.04.2020	Evaluation of COVID-19 in pediatric household contacts of confirmed cases in the KK Women's and Children's Hospital 213 pediatric household contacts; 223 adult index patients	Active assessment and testing of cases and their contacts; admission of positive cases	Positive RT-PCR	RT-PCR testing on nasopharyngeal swabs	0–16; > 16	N.a.	6.13 (2.92; 9.35)
Prazuck et al. (35)	Letter to the Editor	France; 12.03.2020–11.05.2020	Household cluster of 30 members living in a confined environment during the national lockdown; 11; 19	Active cluster investigation	Positive RT-PCR	Clinically examination for all residents, RT-PCR testing for symptomatic cases, rapid serology testing for all subjects >45 days after the symptom onset	2–16; 27–84	22.22 (6.54; 37.90)	N.a.
James et al. (36)	Early Release; Morbidity and Mortality Weekly Report	USA; 06.03.2020–22.04.2020	Events at a Church in Arkansas from 06 to 08.03.2020; 32; 60	Cluster description of a church event with two adult index cases	Positive RT-PCR	RT-PCR; 49% of contacts tested	≤ 18; ≥ 19	38.04 (28.12; 47.96)	N.a.
Li W et al. (37)	Published; Corrected Proof	China; 01.01.2020–01.03.2020	Household clusters each with one index patient with clear history of exposure to Wuhan data, reported in two local hospitals (150 and 250 km from Wuhan); 47 index persons and 64 contacts (4 pediatric contacts included)	Retrospective analysis of hospital records from 2 local hospitals (150 and 250 km from Wuhan) and confirmation/supplementation of data by telephone interviews of household clusters	Positive RT-PCR	RT-PCR testing on nasopharyngeal swabs at beginning and mid of quarantine duration; for symptomatic cases at least 4 times	<18; ≥ 18	16.33 (12.67; 19.99)	N.a.
Park et al. (38)	Online Report	South Korea; 20.02.2020–13.05.2020	Household and non-household clusters defined by contact-tracing; index cases 153; 5553 contacts 694; 58379	Retrospective analysis of the nationwide COVID-19 contact tracing program	Positive RT-PCR	RT-PCR in high-risk contact groups; RT-PCR for symptomatic non-high-risk contacts	0–19; ≥ 20	11.67 (11.05; 12.28)	15.97 (11.74; 20.20)
Kim et al. (39)	Online Report	South Korea; 20.01.2020–06.04.2020	Pediatric index cases and their household members; 355 persons (107 pediatric index included)	Retrospective analysis of the National Notifiable Disease Surveillance System data	Positive RT-PCR; first identified pediatric case or first documented patient within a cluster	RT-PCR for all household contacts	≤ 18; ≥ 19	N.a.	0.40 (0.09; 0.72)
Szablewski et al. (40)	Early Release; Morbidity and Mortality Weekly Report	USA; 17.06.2020–27.06.2020	Overnight camp in Georgia; 509; 88	Cluster description of an overnight camp with one positive staff member (index)	Positive RT-PCR in camp A attendee from a specimen collected or reported to DPH from the first day at camp A	Recommendation for RT-PCR testing for all attendees	6–17; 18–59	N.a.	43.55 (39.57; 47.53)
Danis et al. (41)	Published; corrected version	France, 07.02.2020–End of February	Cluster in a chalet in Contamines-Montjoie; 172 persons	Interview and questionnaires for confirmed cases; daily follow up calls and recommendation for body temperature measurements for low risk contacts	Positive RT-PCR + in–/ direct epidemiological link to the chalet	RT-PCR on nasopharyngeal swabs and endotracheal aspirates; 42% of contacts tested	Not stated	N.a.	0.58 (−0.56; 1.72)

* *Overall Secondary attack rate in close and household contacts, dependent on type of index (adult vs. child) as reported in Figure 7*.

#### Small Contact-Tracing Studies

We identified 12 reports on SARS-CoV-2 transmission, which only described one family or a very small sample of patients and therefore are not included in the meta-analysis (summarized in Table 2) (42–50). While these studies are not suitable for combined analyses, they often report infection clusters in great detail and are therefore useful for understanding transmission patterns despite their small sample sizes.

**Table 2:** Characteristics of studies included in qualitative synthesis, excluded from quantitative review for insufficient data accuracy or small sample size.

**First author**	**Study status; type**	**Country; timing of study**	**Setting**	**n(children); n(adults)**	**Method**	**Case definition**	**Testing**	**Age range (years): child; adult**
Wang Z et al. (26)	Published, Peer Reviewed	China; 13.02.2020–28.02.2020	COVID-19 patients of the Union Hospital in Wuhan City and their household members	Positive cases: 85 Household members: 18; 222	Review of clinical charts and laboratory testing; epidemiological, demographic and symptom data was collected through communication with the index patient or their families	Positive RT-PCR	RT-PCR on throat swabs	Not stated
da Silva et al. (42)	Online Report	Brazil; March–April 2020	Cluster of the first five COVID-19 cases in Tangará da Serra, Mato Grosso	2; 3 (including index cases)	Analysis of documental records and epidemiological investigation of the first cluster	Positive RT-PCR	RT-PCR testing on combined nasopharyngeal and oropharyngeal swabs; Serology testing on serum specimens using rapid test methods	9–12; 35–51
Lin et al. (43)	Online Report	China; 22.01.2020–12.02.2020	Case report of a 7-year old girl admitted to a quarantine ward in local country hospital of Chongqing	2; 3 (including index case)	Report of the clinical presentation of the child case and epidemiological investigation of the transmission chain	Positive RT-PCR	RT-PCR testing on throat swabs; CT chest scans; routine laboratory testing	2–7; not stated
Li C et al. (44)	Online Report	China; 02.02.2020–March 2020	Case report of a 3-month-old child with family contacts who had returned from Wuhan	1; not stated	Report of the clinical presentation of the child case and epidemiological investigation of the transmission chain	Positive RT-PCR	RT-PCR testing on throat swabs; CT chest scans; routine laboratory testing	3 months; not stated
Zhu et al. (45)	Published; Peer reviewed	China; 24.01.2020–22.02.2020	Pediatric case series from 3 hospitals in 3 cities of Jiangsu province	10; 0	Retrospective analysis of medical records from three hospitals	Positive RT-PCR	RT-PCR testing on throat or anal swabs; CT chest scans; routine laboratory testing	19 months−17; none
Pan et al. (46)	Correspondence	China; 22.01.2020–29.01.2020	Case report of a family cluster with two asymptomatic members	1; 2	Description of the cluster regarding the patients' clinical presentation	Positive RT-PCR	RT-PCR testing on nasopharyngeal swabs; CT chest scans; routine laboratory testing	3; 33–35
Mao et al. (47)	Online Report	China; 31.01.2020–29.02.2020	Case report of a 14-month-old boy with household transmitted COVID-19	1; not stated	Report of the clinical presentation of the child case and epidemiological investigation of the transmission chain	Positive RT-PCR	RT-PCR testing on nasopharyngeal swabs; CT chest scans; routine laboratory testing	14 months; not stated
Luo Y et al. (48)	Research Letter	China; 31.01.2020–01.03.2020	Family cluster around a physician in Wuhan	2; 4 (including index case)	Description of the cluster regarding the contacts' clinical presentation	Positive RT-PCR	RT-PCR testing on throat swabs and CT chest scans on all household contacts; RT-PCR on stool specimens, Serology testing and routine laboratory testing	7; 37–64
Zhou et al. (49)	Published; Peer reviewed	China; January–February 2020	Family cluster in the southeast of Zhejiang province	1; 8 (including index case)	Investigation, screening, and medical observation of contacts after reported index case and search for source of infection	Suspected case with positive RT-PCR in respiratory or blood samples	RT-PCR testing on throat swabs for patients and contacts	10; 18–77
Li P et al. (50)	Published; Peer reviewed	China; 26.01.2020–11.02.2020	Family cluster in Wuxi City	1;5 (including index case)	Contact-tracing investigation and description of the cluster	Positive RT-PCR	RT-PCR testing on sputum specimens (and throat swabs)	7; 41–66
Song et al. (51)	Published; Peer reviewed	China; 16.01.2020–06.03.2020	Four family clusters recruited at Bejing Ditan Hospital	8; 16	Retrospective analysis of medical records	Positive RT-PCR	RT-PCR testing on throat swabs; all patients were tested at admission	9 months−10 years; 32–86
Huang et al. (52)	Published; Peer reviewed	China; 23.01.2020–20.02.2020	Cluster of 22 close-contacts of a 22-year-old index patient in Anhui Province; focusing on the symptomatic cases	1;7 (including index case)	Prospective investigation of the close contacts, especially clinical data for the symptomatic contacts	Positive RT-PCR	RT-PCR; CT chest scans; other routine laboratory testing for symptomatic contacts	16; 21–23
Sun et al. (53)	Published; Peer reviewed	China; 28.01.2020–03.03.2020	Family clusters derived from positive children admitted to Wuhan Children's Hospital which was designated for children with COVID-19 infection	Seventy-four infected children	Retrospective case study based on medical records of the Wuhan Children's Hospital focusing on the clinical presentation of the pediatric cases	Positive RT-PCR of one specimen	RT-PCR on nasopharyngeal and anal swabs; CT chest scans; other laboratory testing	1 month−15 years; not stated
Yang et al. (54)	Published, Peer Reviewed	China; 20.01.2020–22.03.2020	Reported cases in Shiyan city identified through the local surveillance program	38; 634	Retrospective analysis of contact tracing reports from the Shiyan Center for Disease Control and Prevention surveillance program and model-based estimation of incubation period and serial interval of COVID-19	Not stated	not stated	<14; ≥ 14
Cai et al. (55)	Published, Peer Reviewed	China; 19.01.2020–03.02.2020	Ten children with confirmed SARS-CoV-2 infection who were admitted to the Children's Hospital in Shangai, Hefei and Qingdao	Ten infected children	Case series of children with COVID-19 focusing on their clinical presentation	Positive RT-PCR on both open reading frame 1ab gene and nucleocapsid protein gene	Duplex RT-PCR on nasopharyngeal and throat swabs; colloidal gold assay for influenza virus A and B on respiratory swabs	3–131 months; none
Li Q et al. (56)	Published, Peer Reviewed	China; December 2019–22.01.2020	First 425 patients with SARS-CoV-2 confirmed pneumonia in Wuhan	0; 425	Identification of suspected cases through the pneumonia of unknown etiology surveillance mechanism and active field investigation by teams of the Chinese Center for Disease Control and Prevention	Positive RT-PCR on both open reading frame 1ab or 1b gene and nucleocapsid protein gene	RT-PCR on upper and lower respiratory tract specimens	<15; ≥ 15
Mizumoto et al. (57)	Preprint, Letter to the Editor	Japan; January–March 2020	Domestically acquired cases	10; 284	Estimation of age-specific attack rates based on retrospective analysis of domestically acquired cases	Positive RT-PCR	RT-PCR	0–19; ≥ 20
Yu et al. (58)	Letter to the Editor	China; 14.01.2020–14.02.2020	Close contacts of various exposure environments to the index cases	249; 1,147	Contact-tracing investigation data and interview data for characterization of close contacts	Not stated	Not stated	0–18; ≥ 18
Rosenberg et al. (59)	Published, corrected proof	USA; March 2020	Positive tested persons in healthcare settings, community-based collection sites and in household setting in New York State	47,326 positive cases	Active cross-sectional study on SARS-CoV-2 prevalence in multiple settings in New York State excluding New York City and epidemiological investigation of cases	Positive RT-PCR	RT-PCR	0–17; ≥ 18
Luo L et al. (60)	Preprint	China; 13.01.2020–06.03.2020	Close contacts of confirmed COVID-19 patients in Guangzhou	783; 4,159	Prospective investigation and characterization of close contacts of confirmed COVID-19 patients using standard questionnaires, symptom monitoring and laboratory testing	Positive RT-PCR	RT-PCR on throat swab samples once every two days	0–17; ≥ 18
Shen et al. (61)	Published, Peer Reviewed	China; 08.01.2020–26.02.2020	Pediatric COVID-19 patients of the Public Health Clinic Center of Changsha	Nine infected children	Single-center study with follow up at the Public Health Clinic Center of Changsha which analyzed epidemiological and clinical data of pediatric COVID-19 cases focusing on clinical presentation of the patients	Positive RT-PCR	RT-PCR, CT chest scans, CT X-ray, other laboratory testing	1–12; none
Ji et al. (62)	Online Report	China; 25.01.2020–not stated	Pediatric patients of two family clusters admitted to Beijing Tsinghua Changgung Hospital	Two infected children	Retrospective review of clinical reports from two family clusters to describe the clinical features of pediatric patients	Positive RT-PCR	RT-PCR on oropharyngeal swabs, CT chest scans, other laboratory testing	9–15; not stated
Posfay-Barbe et al. (63)	Research Briefs	Switzerland; 10.03.2020–10.04.2020	Identification of pediatric patients through the Geneva University Hospital's surveillance network and their household contacts	134 (including 111 pediatric COVID-19 cases); 86	Retrospective review of medical charts and active follow-up of patients and household contacts	Positive RT-PCR	RT-PCR on nasopharyngeal swabs	<16; ≥ 16
Silveira et al. (64)	Preprint	Brazil; 11.04.2020–27.04.2020	Five hundred households of the nine largest cities in the Brazilian state	Four thousand one hundred and eighty-eight tested persons	Two household-based serological surveys in nine of the largest cities of the Brazilian State of Rio Grande do Su	Serology (IgG, IgM)	Serology using the Wondfo lateral flow rapid test	0–19; ≥ 20
Chau et al. (65)	Published, corrected proof	Vietnam; 10.03.2020–05.04.2020	Characterization of quarantined people in Ho Chi Minh City	Thirty participants	Prospective study at a quarantine center for COVID-19 in Ho Chi Minh City collecting epidemiological data and laboratory testing	Positive RT-PCR	RT-PCR on saliva and daily nasopharyngeal/ throat swabs	16–17; 18–60
Liu et al. (66)	Published, Peer Reviewed	China; 15.01.2020–15.03.2020	Clusters identified through contact-tracing in Guangdong Province which was early affected by the pandemic	Confirmed cases 1,361; contacts 1,867; 9,713	Analysis of the dataset provided by the National Internet-Based infectious Disease Reporting System and calculation of COVID-19 attack rates	Positive RT-PCR	RT-PCR on throat swabs	0–19; ≥ 20
Jing et al. (67)	Published, Peer Reviewed	China; 07.01.2020–18.02.2020	Close/household contact clusters identified through contact tracing in Guangzhou	Primary cases 10; 205 contacts; 253; 2,042	Retrospective review of contact tracing dataset from the Guangzhou Center for Disease Control and Prevention and calculation of COVID-19 attack rates	Suspected symptomatic case with positive RT-PCR	RT-PCR on respiratory specimens	<20; ≥ 20
Somekh et al. (68)	Online Report	Israel; not stated	Thirteen family clusters from the city of Bnei Brak	Thirteen family clusters	Analysis of the 13 family clusters regarding intrafamilial transmission chains	Positive RT-PCR	RT-PCR on nasopharyngeal swabs	6 months−17years; 18-48
Zhang W et al. (69)	Research Letter	China; 28.01.2020–15.03.2020	Clusters with presymptomatic or asymptomatic index patients in Guangzhou	Presymptomatic cases 71 persons; close contacts 45, 323	Analysis of contact-tracing surveillance data and calculation of secondary attack rates from different types of contact with presymptomatic patients	Not stated	Not stated	≤ 17; ≥ 18
Bi et al. (70)	Published, Peer Reviewed	China; 14.01.2020–12.02.2020	Confirmed cases identified by the Shenzhen CDC and their close contacts	Three hundred and ninety-one adult COVID-19 cases, 1,286 close contacts	Comparison of cases identified through symptom-based surveillance and contact tracing	Positive RT-PCR	RT-PCR on nasal swabs	0–19; ≥ 20
COVID-19 National Emergency Response Center (71)	Published, Peer Reviewed	South Korea; 24.01.2020–10.03.2020	Reported cases and their close and daily contacts	30 first cases, traced contacts 155; 2201	Retrospective review of COVID-19 reporting and surveillance data from Korea Centers for Disease Control and Prevention	Not stated	Not stated	0–19; ≥ 20
Chaw et al. (72)	Preprint	Brunei Darussalam; 28.02.2020-02.04.2020	Secondary cases with a link to the Malaysian Tablighi Jama'at religious gathering cluster with 19 positive cases	14; 57	Retrospective analysis from digital inpatient records on the national health information system database which were completed by oral histories	Positive RT-PCR on nasopharyngeal swab	RT-PCR on nasopharyngeal swabs	9 months−17 years; 20–68
Cheng et al. (73)	Published, Peer Reviewed	Taiwan; 15.01.2020-02.04.2020	Laboratory-confirmed cases and their close contacts	One hundred confirmed patients; Close Contacts 281; 2,286	Prospective case-based study of confirmed cases and their close contacts with active follow up until 14 days after last exposure to the index case	Positive RT-PCR	RT-PCR for high-risk contacts and for symptomatic close contacts	0–19; ≥ 20
Dattner et al. (74)	Preprint	Israel, January 2020–02.05.2020	Household clusters derived from contact-tracing	3,353 people	Estimation of the relative susceptibility and infectivity of children based on a discrete stochastic dynamic model derived from data of the Israeli and municipality COVID-19 database	Positive RT-PCR	RT-PCR for all household members	0–20; 20–100
Wolf et al. (75)	Online Report	Germany; 24.01.2020–not stated	Clinical and virological characterization of 3 children from one of the first family clusters in Munich	3; 2	Characterization of the family cluster regarding transmission details, epidemiological data and focus of the clinical presentation of the three child cases	Positive RT-PCR	RT-PCR on nasopharyngeal, stool and blood specimens, virus culture from nasopharyngeal swabs, other laboratory testing	7 months−5 years; not stated
Su et al. (76)	Published, Peer Reviewed	China; 24.01.2020–24.02.2020	Family clusters with children infected after their family's onset who were admitted to the Jinan Infectious Disease Hospital	9; 14	Retrospective review of clinical records and laboratory testing	Positive RT-PCR	RT-PCR on nasopharyngeal swabs, sputum, and stool specimens; testing for other virus and bacteria; CT chest scans; other laboratory testing	11 months−9 years; 30–72
Chan et al. (77)	Published, Peer Reviewed	China; 26.12.2019–15.01.2020	Family cluster with five cases of initially unexplained pneumonia after a visit to Wuhan	infected cases 1; 5	Case report with analysis of history, physical findings, and laboratory investigations	Positive RT-PCR	RT-PCR on respiratory, stool, serum, or plasma samples; whole-genome sequencing and phylogenetic tree construction; CT chest scans	10; 36–66
Wei M et al. (78)	Research Letter	China; 08.12.2019–06.02.2020	Hospitalized infants infected with SARS-CoV-2	Nine infected children	Retrospective analysis of surveillance records from hospitalized infants diagnosed with COVID-19 with focus on clinical presentation and epidemiologic history	Positive RT-PCR	RT-PCR on nasopharyngeal swabs	1 month−11 months; none
Jiang et al. (79)	Letter to the Editor	China; 23.01.2020–13.02.2020	Case report of two households with one index patient	1; 12 (including index case)	Retrospective analysis of information provided by the Infection Department of Changyuan People's Hospital	Positive RT-PCR	RT-PCR, CT chest scans	9; 34–87
Wu et al. (80)	Published, Peer Reviewed	China; 20.01.2020–27.02.2020	Pediatric patients with SARS-CoV-2 of two Hospitals in Qingdao and Wuhan	Seventy-four pediatric COVID-19 patients	Retrospective analysis of electronic medical records from pediatric patients admitted to two Children's Hospitals in Qingdao and Wuhan	Positive RT-PCR on nasopharyngeal swabs	RT-PCR on nasopharyngeal swabs, CT chest scans, other laboratory testing	≤ 3 months–>10 years; none
Chen et al. (81)	Online Report	China; 01.02.2020–03.02.2020	One familial cluster with 2 infected children	2; 2	Report of the epidemiological and clinical characters of a familial cluster with pediatric cases and linkage to Wuhan	Positive RT-PCR	RT-PCR, CT chest scans, other laboratory testing	8–9; 34–not stated
Maltezou et al. (82)	Published, Peer Reviewed	Greece; 26.02.2020–03.03.2020	Family clusters with at least one child identified through the national registry of SARS-CoV-2 infections	43; 66	Retrospective analysis of surveillance records regarding disease progress and transmission chains from family clusters with one child and SARS-CoV-2 infection who were diagnosed by two laboratories in Athens and one in Thessaloniki	Positive RT-PCR	RT-PCR on respiratory samples and testing for viral load	10 days−17 years; 18–76

Song et al. (51) investigated four family clusters with intrafamilial SARS-CoV-2 transmission. All four index patients were adults, exposing 24 household contacts. The SAR for adult contacts was 92% (11 of 12 exposed adults) and 58% (7 of 12) for exposed children, indicating a lower susceptibility of children.

Huang et al. (52) prospectively traced close contacts of a 22 year-old young man. Though being asymptomatic, the young adult spread the virus effectively as proven by a high SAR among the exposed. The index patient met with his 16 years old cousin and uncle for dinner and moved on to meet 15 previous classmates, all aged 22 years, for a classmate-get-together. The 16 years old cousin and 6 of 16 exposed adults were tested positive for SARS-CoV-2 by RT-PCR.

#### Contact-Tracing Studies Without Detailed Information on Transmission Chains

We identified 28 studies with data on transmission of SARS-CoV-2 in settings of close contacts and households, but the exact description of transmission chains remained elusive in many of the studies (summarized in Table 2) (26, 53–80). Nevertheless, several studies contain relevant information on susceptibility and contagiousness of children.

Posfay-Barbe et al. (63) conducted a study on all patients younger than 16 years with SARS-CoV-2 infection in the Geneva University Hospital's surveillance network. Among 4,310 patients with SARS-CoV-2 infection reported to the Geneva University Hospital's surveillance network, only a small proportion of 40 were younger than 16 years (0.9%). Among those, 39 children and 111 household contacts could be included for further studies and interviews. In 31 of 39 (79%) of infected children, household contacts were suspected or confirmed with COVID-19 before the study child. In 8% (3 of 39) of cases, the child developed symptoms first. No secondary cases in household contacts of a child index cases was identified. This study illustrates how difficult it is to establish exact patterns of transmission for viral infections with mild symptoms in children.

In a large retrospective analysis from China, Liu et al. (66) enrolled 11.580 contacts of 1,361 COVID-19 cases from January to March 2020. Contacts were clinically evaluated and tested by RT-PCR for SARS-CoV-2 from throat swabs by the Chinese Centers for Disease Control. Contacts remained quarantined and swabs were repeatedly taken. The SARs were 5.7% for children of 0–9 years (60 of 1,048) and 4.0% (33 of 819) for children of 10–19 years.

Jing et al. (67) analyzed a comprehensive dataset of household contacts and residential data to calculate SARs. Between January and February 2020, data from 215 primary cases, 134 secondary or tertiary cases, and 1,964 uninfected close contacts were evaluated, and a SAR of 17.1% (13.3–21.8) was estimated. The SAR among children with an adult was 5.2% (2.4–9.7%) for household contacts and 1.4% (95%CI 0.04–7.6) for non-household contacts.

Somekh et al. (68) identified 13 family clusters of infection in the city Bnei Brak in Israel before May 2020. Children were analyzed in two groups, younger than 5 years or between 5 and 18 years. Of the children younger than 5 years, 2 of 18 (11.8%), and of those older than 5 years, 13 of 40 (32.5%) were tested positive for SARS-CoV-2 by RT-PCR. Adults (>18 years of age) were tested positive in 21 of 36 cases (58.3%). Thus, in household settings with a COVID-19 patient, the authors found that children aged 0–4 years were 47% and children aged 5–17 years were 61% less likely to have a positive PCR result as compared to adults living in the same household.

Zhang et al. (69) investigated 369 close contacts of RT-PCR -positive index persons in Ghuangzhou, China. Among the contacts the SAR in children aged younger than 18 years was 4.3% (95%CI 1.2–14.5), which was higher than in adults aged 31–40 years (1.4%, 0.2–7.4%) but lower than in the elderly above 60 years of age (8.0%; 1.4–27.5%).

Bi et al. (70) retrospectively analyzed data obtained from Shenzhen CDC between January 14th and February 9th 2020. 1,286 close contacts were identified, among them 148 children younger than 9 years and 85 aged 10–19 years. SAR for children under 9 years were 7.4% (95%CI 4.2–12.8) and 7.1% (3.3–14.6%) for children aged 10–19 years.

Chaw et al. (72), respectively investigated household SARs of a cluster in Brunei Darussalam. All contacts and household members of confirmed infections were tested by RT-PCR of nasal swabs. An overall household attack rate of 10.6% (95% 7.3–15.1%) was estimated. Attack rates for spouses was higher (41.9%; 24.1–60.7%) than for children (14.1%, 7.8–23.8%).

In a modeling approach from the city of Bnei Brak (Israel), Dattner et al. (74) aimed to estimate relative susceptibilities of children vs. adults in a household setting. They estimate that the relative susceptibility of children for a SARS-CoV-2 infection is 43% (95%CI 31%, 55%) of the susceptibility of adults, and that the relative contagiousness of children is 63% (95%CI 37%, 88%) of the infectivity of adults.

These studies were not included meta-analysis because of an often unclear index patient and the enormous heterogeneity of the study design. In summary, these discussed studies confirm children to show a milder disease course and to have a lower seroprevalence indicative of a lower susceptibility to infection with SARS-CoV-2. However, the data regarding the transmission risk from an infected child are partially contradictive.

#### Seroprevalence Studies

We included 7 seroprevalence studies from 6 countries worldwide (summarized in Table 3). Two studies were conducted in France and one in Chile, Germany, Spain, Switzerland, and the USA, respectively. While these studies were not designed to address transmission patterns, they are among the largest cohort studies to date and therefore provide robust information on the age-dependent infection risk with SARS-CoV-2.

**Table 3:** Characteristics of study results from PCR-prevalence and serology studies.

**First author**	**Study status; type**	**Country; timing of study**	**Setting**	**n(children); n(adults)**	**Method**	**Case definition**	**Testing**	**Age range (years): child; adult**
Streeck et al. (27)	Preprint	Germany; 31.03.2020–06.04.2020	Random household-based study population of Gangelt six weeks after a super-spreading event	61; 858	Cross-sectional epidemiological study based on a random sample of 600 inhabitants using laboratory testing methods and questionnaire-based information	Serology (IgG)	RT-PCR testing on pharyngeal swabs; serology using an enzyme-linked immunosorbent assay	
Fontanet et al. (28)	Preprint	France; 30.03.2020–04.04.2020	Students and their houshold members and staff of a high school located in Oise, a department heavily affected by the pandemic	37; 623	Retrospective closed cohort study using questionnaires and blood testing	Serology (IgG)	Serology using several assays developed by Institute Pasteur (N assay, S-Flow assay, LIPS assay)	≤ 17; ≥ 18
Pollán et al. (29)	Published, Peer Reviewed	Spain; 27.04.2020–11.05.2020	National representative sample from randomly selected households of municipalities acrouss the country	11,422; 49,653	Nationwide cross-sectional epidemiological study based on a random sample, using laboratory testing and questionnaire-based information	Serology (IgG)	Serology using a lateral flow immunochromatographic assay and a chemiluminescent microparticle immunoassay	0–19; ≥ 20
Stringhini et al. (30)	Published, Peer Reviewed	Switzerland; 06.04.2020–09.05.2020	Household-based study population of the canton of Geneva with the same age distribution	455; 2311	Population-based study of former participants of a yearly representative stratified sample for the Bus Santé study, a cross-sectional health assessment study	Serology (IgG)	Serology using an enzyme-linked immunosorbent assay and recombinant immunofluorescence assay for potentially indeterminate individuals and all positives	5–19; ≥ 20
Laxminarayan et al. (32)	Preprint	India; 05.03.2020–04.06.2020	Clusters defined by contact-tracing in the states Tamil Nadu and Andhra Pradesh	Tamil Nadu 42,506; 476,429 tested; Andhra Pradesh 30,076; 446,912 tested	Retrospective analysis of the surveillance program including contact tracing data	Positive RT-PCR	Initially RT-PCR for symptomatic individuals with history of travel or contact of confirmed case; expanded to all symptomatic individuals and asymptomatic contacts of confirmed cases between 20.-28.03.2020	0–17; ≥ 18
Torres et al. (83)	Published, Peer Reviewed	Chile; 04.05.2020–19.05.2020	Random selection of students and staff of a school community outbreak in Stantiago nine days after the first country case	1,009; 235	Cross-sectional epidemiological study based on at home sampled specimens and a web-based questionnaire	Serology (IgG, IgM)	Serology using the IgG/IgM Test Kit (Colloidal gold) from Genrui Biotech Inc.	Pre-school–High School Students; not stated
Bendavid et al. (84)	Preprint	USA; 03.04.2020–04.04.2020	Community sample drawn from Santa Clara County, the county with the largest number of confirmed cases in Northern California at time of study	621; 2709	Cross-sectional epidemiological study based on a Facebook derived cohort targeting the specific sociodemographic characteristics of the county	Serology (IgG, IgM)	Serology using a lateral flow immunoassay	0–18; ≥ 19
Cohen et al. (85)	Preprint	France; 14.04.2020–12.05.2020	Children consulting an ambulatory pediatrician in the Paris area (most affected region during the pandemic)	Six hundred and five children	Cross-sectional prospective multicenter study testing symptomatic and pauci- children who consulted an ambulatory pediatrician	Positive RT-PCR, Serology (IgG, IgM)	RT-PCR testing on nasopharyngeal swabs; micro-method serology using a rapid chromatographic immunoassay	0–15; none
Stein-Zamir et al. (86)	Rapid Communication	Israel; 26.05.2020–mid-June 2020	High school cluster 10 days after schools' reopening with two independent index cases	1,164; 152	Epidemiological investigation of the high school outbreak and comparison of the age distribution of COVID-19 cases in the Jerusalem district vs. The rest of the country	Positive RT-PCR	RT-PCR testing on nasopharyngeal swabs	7th to 12th grade students; not mentioned
Gudbjartsson et al. (87)	Published; Peer reviewed	Iceland; 31.01.2020–04.04.2020	Persons living in Iceland with high risk for infection (e.g. due to travel history or exposure to confirmed cases) and population screening	848; 12,232	Targeted testing of persons with high risk for infection and population screening using an open invitation in the first round and using invitation of a random sample in the second round	Positive RT-PCR	RT-PCR testing on naso- and oropharyngeal swabs; multiplex PCR; Sequencing	0–9; ≥ 10
Lavezzo et al. (88)	Preprint	Italy; 21.02.2020–29.02.2020	Person living in the municipality of Vo', which was early affected by the pandemic, during the 14-day lockdown	467; 2,345	Epidemiological investigation at start and mid of lockdown using laboratory testing, reconstruction of transmission chains and recording of symptoms	Positive RT-PCR	RT-PCR testing on nasopharyngeal swabs	0–20; ≥ 21

Torres et al. (83) investigated a SARS-CoV-2 outbreak in a school in Santiago, Chile, where one teacher and another member of staff tested positive for SARS-CoV-2 by RT-PCR early during the pandemic, when the whole school was put under quarantine. Eight to ten weeks later, students, parents and all staff were evaluated for SARS-CoV-2 serology. Overall, the antibody positivity was higher in staff (16%, 95%CI 12.1; 21.9; 39 of 235) than in students (10%, 95%CI 8.2–11.8%; 100 of 1,009), i.e. they were in contact with young students.

Stringhini et al. (30) conducted study a investigating the seroprevalence in a cohort representative for the canton of Geneva. They found a remarkably lower seroprevalence in children younger than 9 years (0.8%; 1 of 123), than in children aged 10–19 years (9.6%, 32 of 332) or adults aged 20–49 years (9.9%, 108 of 1,096), indicating a decreasing susceptibility of infection with decreasing age.

A similar picture was found in a study on a school outbreak in France (28). The authors defined the infection attack rate (IAR) as the proportion of participants testing positive for SARS-CoV-2 antibodies. IAR for children younger than 14 years was 2.7% (1 of 37), whereas children aged 15–17 years showed an IAR of (82 of 205). The IAR for parents of students and siblings of students were 11.4% (24 of 211) and 10.2% (13 of 127), respectively, indicating a lower susceptibility of infection in children younger than 14 years and likely lower contagiousness in children aged 15–17 years, as their parents and sibling had a lower IAR.

However, this age dependence of seropositivity for SARS-CoV-2 antibodies could not be found in all countries: Bendavid et al. (84) conducted a cross-sectional study in Southern California, USA to get an estimate of SARS-CoV-2 seroprevalence on April 3rd to 4th 2020. The overall prevalence of antibodies was 1.5% with a negligible differences between age groups (0–4 years 1.4%, 5–18 years 1.5%, 19–64 years 1.5%, >65 years 1.2%).

One of the largest published seroprevalence studies from Spain by Pollán et al. (29) comprised more than 61.000 participants. They found the lowest positivity for antibodies against SARS-CoV-2 in infants younger than 1 year (1.1%, 95%CI 0.3-3.5), followed by children aged 5-9 years (3.1%, 95%CI 2.2-4,2). The overall seropositivity in children younger than 19 years was lower (3.4%, 95%CI 2.9-3.9) than in adults aged 35 to 49 years (5.3%, 95%CI 4.7–5.9). Another important finding of this study was the robust performance of a lateral flow assay for SARS-CoV-2 serology, with only relatively small differences to antibody titers measured by ELISA.

To the contrary, Cohen et al. (85) conducted a cross-sectional prospective multicenter study from April 14th to May 12th in France with a comparatively high seropositivity for SARS-CoV-2 antibodies of 10.7% among all children.

Streeck et al. (27) undertook a cross-sectional study in a community during a super-spreading event in North Rhine-Westphalia, Germany from March 31st to April 6th 2020. They found no statistically significant differences in the rate of infection associated with age or sex, but overall children below the age of 14 years had a lower infection rate than adults.

#### PCR Prevalence Studies

We identified 4 PCR-prevalence studies worldwide, one each from France, India, Israel, Iceland, and Italy (summarized in Table 3).

Laxminarayan et al. (32) investigated the disease surveillance data collected through June 4th 2020 from the provinces Tamil Nadu and Andhra Pradesh in India, resulting in one of the largest PCR-based studies on SARS-CoV-2 to date. A total of 33.584 RT-PCR confirmed cases of SARS-CoV-2 infection were included in the analysis. SAR estimates ranged from 1.0% (95%CI 0.0–5.4) in healthcare settings to 2.6% (95%CI 1.6–3.9) in the community and 9.0% (95%CI 7.5–10.5) in the household. Overall, 48.3% of all positive contacts were traced to an index case in their household. While contacts of index cases, who were children appeared more likely to be infected than contacts of adult index cases, this pattern did not persist after adjusting for the fact that contact with children more often occurred in household settings.

Stein-Zamir et al. (86) retrospectively investigated a local SARS-CoV-2 outbreak in a high-school in Israel just after students returned to school in May 2020. A total of 151 staff members and 1,161 students were tested after 2 students from different grades were tested positive for SARS-CoV-2 by RT-PCR. The attack rate defined as individuals with positive RT-PCR of all tested individuals was 13.2% for the students and 16.6% for members of staff. COVID-19 rates were higher in junior grades (7–9) than in high grades (10–12).

In order to characterize the spread of COVID-19 in Iceland, Gudbjartsson et al. (87) performed a targeted testing among returning travelers as well as population screening of randomly invited individuals. They found that children under 10 years of age were less likely to receive a positive result than were persons 10 years of age or older, with percentages of 6.7 and 13.7%, respectively, for targeted testing; in the population screening, no child under 10 years of age had a positive PCR result, as compared with 0.8% of those 10 years of age or older. Specifically of the 564 children under the age of 10 years in the targeted testing group, 38 (6.7%) tested positive, in contrast to positive test results in 1,183 of 8,635 persons, who were 10 years of age or older (13.7%). None of the 848 children under the age of 10 years tested positive, as compared with 100 of 12,232 persons (0.8%; 95%CI, 0.7 to 1.0) 10 years of age or older.

Lavezzo et al. (88) studied the SARS-CoV-2 outbreak in the municipality of Vo' in Italy by conducting RT-PCR at two time-points. They found no relevant differences in viral load of symptomatic and asymptomatic infections. No infections were detected in either survey in 234 tested children ranging from 0 to 10 years of age, including those living in the same household as infected individuals. These include at least 13 children, who lived in one household with an infected family member.

#### Studies on Viral Loads in Children Compared to Adults

Several groups investigated the nasopharyngeal SARS-CoV-2 viral load (VL) of infected children as a correlate of contagiousness (65, 75, 89–95), summarized in Supplementary Table 1. A German preprint that was intensely discussed mainly because of questions regarding the statistical data interpretation found no significant VL differences between children and adults and therefore cautioned against “an unlimited re-opening of schools and kindergartens in the present situation” – a conclusion that was weakened in the revised version of the preprint manuscript (96). Similar VL in swabs from children and adults were also found in a recent swiss study (89) comparing VLs from 59 children and adolescents to that of 346 adults. Looking at a small cohort of 12 children the same group also showed that culture-competent SARS-CoV-2 could be isolated from infected children as young as 7 days old (90). SARS-CoV-2 RNA has also been detected in fecal samples of infected children at high frequencies – also if nasopharyngeal swabs where negative – with viral persistence exceeding 70 days (94). However, the role of fecal-oral transmission for the spread of COVID-19 remains unclear.

In summary, nasopharyngeal VLs of SARS-CoV-2 infected children and adults seem to be comparable and infectious virus can also be isolated from the upper airways of neonates.

## Discussion

We identified a rapidly growing body of literature on the transmission risks and transmission patterns for SARS-CoV-2 infections in the general population. Data on transmission patterns in children and young adults and infections in households and close contacts was relatively scarce and revealed partially contradicting results.

### Summary of Evidence

#### General Remarks

It is now well-established that children infected with SARS-CoV-2 have relatively mild symptoms and a favorable course of disease. Few deaths in children with underlying conditions such as inborn or acquired immunodeficiencies or iatrogenic immunosuppression due to solid organ transplantation have been reported, but absolute and relative numbers are much lower than in adults. Especially the significant fraction of asymptomatically infected children makes it difficult to identify child index patients in SARS-CoV-2 infection clusters and to unequivocally define transmission chains.

#### Susceptibility for SARS-CoV-2 Infection in Children

Summarizing the seroprevalence studies, the picture remains heterogenous. population of children in detail, the studies of Stringhini (30), Fontanet (28), Streeck (27), and Pollán (29) found lower antibody positivity in children aged younger than 10 years when compared to the older children aged 10–20 years. No differences existed in antibody prevalence in the study conducted by Bendavid (84), where all age groups were around a rate of 1.5% positive.

The body of evidence that comes from PCR prevalence testing shows a similar picture. Gudbjartsson et al. (87) found that children under the age of 10 years had a lower proportion of positive PCR than older children or adults. This finding is supported by the study from Lavezzo (88), where none of 234 tested children was positive on nasopharyngeal swab for SARS-CoV-2 PCR including 13 children living in one household with adults tested positive for SARS-CoV-2.

Overall there is preliminary evidence from the seroprevalence studies and population-based PCR studies that children have a lower susceptibility to SARS-CoV-2 than adults. As all of the studies were conducted when contact restrictions for children such as school closures were active, the lower seroprevalence is likely influenced by a reduction in exposure. While this is a clear limitation, the effect size of these measures is currently unknown and unlikely fully explains the worldwide lower rate of children infected with SARS-CoV-2. Of note, the lower susceptibility of children compared to adults is a continuum, and the break point at which children show a comparable susceptibility is currently unknown.

#### Contagiousness of Children

We performed a meta-analysis of selected studies in order to compare the risk of SARS-CoV-2 transmission from children vs. adults. As discussed, the data is predominantly derived from small sample size studies, local outbreaks, or contact tracing in single families. When only selecting studies with detailed information on transmission pathways, we could not find evidence for a lower transmission risk arising from an infected child. This would be in concordance with studies finding comparable viral loads in children vs. adults. However, when also taking into consideration that we excluded 2 studies where pediatric index cases did not lead to secondary cases, children may be less infectious than adults. But one caveat remains when addressing this question: With children being mostly pauci- to asymptomatic, they might be missed as a true index patient of an infection cluster. Finally, the age-specific susceptibility to infection with SARS-CoV-2 is introducing another layer of complexity to this burning and complex question as discussed above.

Nevertheless, the findings that (1) few outbreak clusters have been reported from preschools and kindergartens and (2) the lower positivity rates of SARS-CoV RT-PCRs in children compared to adults during the current second infection wave in Europe – despite open schools at the beginning of the infection wave in many European countries-support the notion that especially young children cannot be viewed as drivers of the pandemic.

### Limitations

The COVID-19 pandemic generates scientific knowledge on a single disease at an unprecedented speed. However, especially early in the pandemic, criteria, and definitions for the disease caused by SARS-CoV-2 were uncertain. Accordingly, we found very heterogenous disease definitions, especially in children, including diagnosis based on imaging including serial chest CT scans, PCR detection of SARS-CoV-2, SARS-CoV-2 serology, or just a set of clinical signs. This heterogeneity may in part explain the contradictory results we found in our study. Moreover, the contact restrictions including school closures influence all epidemiological studies by unknown effect sizes.

## Conclusions

Robust data on transmission patterns in households are scarce. Especially younger children seem to be less frequently infected with SARS-CoV-2 compared to adults and can therefore currently not be viewed as drivers of the pandemic. In contrast to the susceptibility, the individual contagiousness of an infected child is a lot more difficult to analyze. Our meta-analysis, which needs to be interpreted with caution did not show a significantly different SAR from children vs. adult index cases. Of note, the current body of evidence regarding the infectivity is of great heterogeneity both in quality and conclusions drawn.

To improve our knowledge on SARS-CoV-2 transmission patterns in children, and thus their contribution to the COVID-19 pandemic, we urgently require sufficiently large studies of high quality. To get there, improved study designs of household studies should include (1) the stringent selection of cases where a true index patient bringing the infection into the household is known (2) all household members are monitored by serial PCR or rapid antigen tests during the quarantine period (3) hygiene measures during the quarantine period are investigated (4) all household members are followed up serologically after the infectious period. Comparing a sufficient number of pediatric and adult index case households with this obviously laborious prospective study design should help to improve the urgently needed better understanding of transmission patterns of SARS-CoV2 between children and adults.

## Data Availability Statement

The original contributions presented in the study are included in the article/Supplementary Material, further inquiries can be directed to the corresponding author/s.

## Author Contributions

BS and TG designed the study, selected literature, and extracted data. BS was the primary author of the manuscript, together with RE. TG designed tables and figures. AG conducted the meta-analysis. All authors contributed to the article and approved the submitted version.

## Conflict of Interest

The authors declare that the research was conducted in the absence of any commercial or financial relationships that could be construed as a potential conflict of interest.

## Footnotes

^1^https://www.who.int/emergencies/diseases/novel-coronavirus-2019/technical-guidance/naming-the-coronavirus-disease-(covid-2019)-and-the-virus-that-causes-it.
