# Supplementary material for: Intra-Household and Close-Contact SARS-CoV-2 Transmission Among Children – a Systematic Review
Source: Front Pediatr. 2021 Apr 9;9:613292. doi: 10.3389/fped.2021.613292 (PMC8062727; doi:10.3389/fped.2021.613292)
Supplement: Supplementary file 1 [file Data_Sheet_1.PDF]

```
#Cases per population per country####
setwd("C:/Users/User/Documents/Uni/Forschungsprojekte/Review_SARS2_houshold (Tessa)")

data_covid_countries <- read.csv("WHO-COVID-19-global-data.csv", sep = ",", header = TRUE, encoding = "UTF-8-BOM")

colnames(data_covid_countries)[1] <- "Date_reported"
data_covid_countries$Date_reported <- as.Date(data_covid_countries$Date_reported)

Country <- c("Germany", "France", "Singapore", "United States of America", "Republic of Korea",
             "Australia", "Brazil", "Chile", "China", "Finland", "Greece",
             "India", "Ireland", "Israel", "Switzerland", "The United Kingdom", "Viet Nam",
             "Italy", "Spain", "Iceland", "Brunei Darussalam", "Japan")
Country_population <- c(83783945, 65273512, 5850343, 331002647, 51269183,
                        25499881, 212559409, 19116209, 1439323774, 5540718, 10423056,
                        1380004385, 4937796, 8655541, 8654618, 67886004, 97338583,
                        60461828, 46754783, 34125, 437483, 126476458)
cp <- cbind.data.frame(Country, Country_population)

newtable <- merge(data_covid_countries,cp, by  = "Country")
newtable$Country <- as.character(newtable$Country)

library(dplyr)
newtable <- newtable %>%
  mutate(New_cases_per_pop = New_cases/(Country_population/100000))
newtable$Country <- replace(newtable$Country, which(newtable$Country == "The United Kingdom"), "United Kingdom")
newtable$Country <- replace(newtable$Country, which(newtable$Country == "Republic of Korea"), "South Korea")
newtable$Country <- replace(newtable$Country, which(newtable$Country == "United States of America"), "United States")
newtable$Country <- replace(newtable$Country, which(newtable$Country == "Viet Nam"), "Vietnam")

#Datatable of studies####
df_master <- read.csv("Mastertable_included references_copy_240820_short.csv", sep = ";", header = TRUE, encoding =
"UTF-8-BOM")

df_master$Study_start[which(df_master$Study_start == "N/a")] <- NA
Date_of_study <- as.Date(as.character(df_master$Study_start),format="%d.%m.%Y")
study_name <- as.character(df_master$Title)
annotation_letter <- as.character(df_master$annotation_letter)
First_author2 <- as.character(df_master$First.Author)
First_author3 <- gsub("\\ .*","",First_author2)
First_author <- paste0(First_author3, " et al.")
Country_4 <- replace(as.character(df_master$Country.of.study),which(as.character(df_master$Country.of.study) == "The
United Kingdom"), "United Kingdom")
Country_4 <- replace(Country_4,which(Country_4 == "Republic of Korea"), "South Korea")
Country_4 <- replace(Country_4,which(Country_4 == "Viet Nam"), "Vietnam")
Country_2 <- replace(Country_4,which(Country_4 == "United States of America"), "United States")
Height <- vector(mode="numeric", length=length(Country))

for(i in c(1:length(Country_2))){
  if(max(newtable$New_cases_per_pop[which(newtable$Country == Country_2[i])]) != 0){
    Height[i] <- max(newtable$New_cases_per_pop[which(newtable$Country == Country_2[i])])
  }else{
    Height[i] <- 0
  }
}
df1 <- cbind.data.frame(Date_of_study, study_name, annotation_letter,Height, Country_2, First_author)

colnames(df1) <- c("Date_of_study", "study_name", "annotation_letter", "Height","Country", "First_author")
df1 <- df1[-which(is.na(df1$Date_of_study) == TRUE),]


#Data Covid-19  Lockdown measures####
data_covid_lockdown_countries <- read.csv("OxCGRT_latest.csv", sep = ",", header = TRUE, encoding = "UTF-8-BOM")
data_covid_lockdown_countries$Date <- as.Date(as.character(data_covid_lockdown_countries$Date),format="%Y%m%d")

df_lockdown_red <- data_covid_lockdown_countries[which(data_covid_lockdown_countries$CountryName %in% c(Country_2,
"United States", "United Kingdom", "Vietnam", "South Korea")),]

Country_3 <- unique(df_lockdown_red$CountryName)

library(tidyr)
df2 <- df_lockdown_red

df2 <- df2[,-c(2,5,7,9, 11, 13, 15, 17, 19:26, 28:42)]

df2 <- df2 %>%
  gather("measures", "value", -Date, -CountryName)

#Plots####

library(ggplot2)
library(RColorBrewer)
library(ggpubr)

annotation_text <- paste0(
  "Description: The graph at the top of the page showed the number of new cases of SARS-CoV-2 infected persons per
```

```
100.000 population between January and August 2020. ",
  "Every included study with a given starting date was highlighted by a vertical marker and could be found more ",
  "detailed in the table below. Additionaly, we displayed a set of Government response indicators in a heatplot
derived from the Oxford ",
  "COVID-19 Government Response Tracker Dataset by Hale et al. Though, higher numbers are indicating a more ",
  "strict response by the Government for the given part of social live.") %>%
  paste(collapse = "\n")

text_annotation <- ggparagraph(text = annotation_text, face = "italic", size = 11, color = "black")

text_annotation1 <- ggplot()

for(i in c(1:length(Country_3))){
  assign(paste0("df1_",Country_3[i]), filter(df1, Country == paste0(Country_3[i])))
  assign(paste0("df2_",Country_3[i]), filter(df2, CountryName == paste0(Country_3[i])))
  assign(paste0("df3_",Country_3[i]), filter(newtable, Country == paste0(Country_3[i])))

  if(exists(paste0("df2_", Country_3[i]))){
    assign(paste0("heatmap_measures_", Country_3[i]),
           ggplot(data = get(paste0("df2_",Country_3[i])),
                  aes(x = Date, y = measures, fill = as.factor(value)))+
             geom_tile()+
             scale_y_discrete(limits = rev(unique(get(paste0("df2_", Country_3[i]))[,3])))+
             scale_x_date(limits = as.Date(c("2020-01-01", "2020-08-10")), breaks = as.Date(c("2020-01-01", "2020-02-
01", "2020-03-01", "2020-04-01", "2020-05-01",
                                                                                               "2020-06-01","2020-07-
01","2020-08-01")),
                          labels = c("Jan", "Feb", "Mar", "Apr", "May", "Jun", "Jul", "Aug"))+
             scale_fill_brewer(name = "level of measures", palette = "RdYlGn", limits = c(4,3,2,1,0))+
             theme_classic()+
             theme(legend.position = "bottom")
    )
  }
  if(exists(paste0("df1_", Country_3[i]))&exists(paste0("df3_", Country_3[i]))&Country_3[i]%in%df1$Country){
    assign(paste0("cases_plot_", Country_3[i]),
           ggplot()+
             geom_line(data = get(paste0("df3_", Country_3[i])), aes(x = Date_reported, y = New_cases_per_pop, color
= Country), size=0.8)+
             labs(y = "New cases per 100.000 population", x = "Date")+
             scale_x_date(limits = as.Date(c("2020-01-01", "2020-08-10")), breaks = as.Date(c("2020-01-01", "2020-02-
01", "2020-03-01", "2020-04-01", "2020-05-01",
                                                                                               "2020-06-01","2020-07-
01","2020-08-01")),
                          labels = c("Jan", "Feb", "Mar", "Apr", "May", "Jun", "Jul", "Aug"))+
             theme_bw()+
             geom_text(data = get(paste0("df1_", Country_3[i])), aes(x = as.Date(Date_of_study), y = (Height +
Height*0.2)), label = get(paste0("df1_", Country_3[i]))[, c("annotation_letter")])+
             geom_segment(data = get(paste0("df1_", Country_3[i])), aes(x = as.Date(Date_of_study), xend =
as.Date(Date_of_study), y = 0, yend = Height))+
             theme(legend.position = "none")
    )
  }else{
    assign(paste0("cases_plot_", Country_3[i]),
           ggplot()+
             geom_line(data = get(paste0("df3_", Country_3[i])), aes(x = Date_reported, y = New_cases_per_pop, color
= Country), size=0.8)+
             labs(y = "New cases per 100.000 population", x = "Date")+
             scale_x_date(limits = as.Date(c("2020-01-01", "2020-08-10")), breaks = as.Date(c("2020-01-01", "2020-02-
01", "2020-03-01", "2020-04-01", "2020-05-01",
                                                                                               "2020-06-01","2020-07-
01","2020-08-01")),
                          labels = c("Jan", "Feb", "Mar", "Apr", "May", "Jun", "Jul", "Aug"))+
             theme_bw()+
             theme(legend.position = "none")
    )
  }
  df_study_print <- get(paste0("df1_",Country_3[i]))[, c("annotation_letter", "First_author", "Date_of_study")]
  colnames(df_study_print) <- c("Annotation", "Authors", "Date of Study")
  df_study_print <- df_study_print[order(df_study_print$`Date of Study`),]
  if(nrow(df_study_print) != 0){
    assign(paste0("study_table_", Country_3[i]),
           ggtexttable(df_study_print, rows = NULL, theme = ttheme("classic"))
    )
  }
  if(nrow(get(paste0("df3_", Country_3[i])))!= 0){
    assign(paste0("total_plot_", Country_3[i]), ggarrange(get(paste0("cases_plot_", Country_3[i])) ,
get(paste0("heatmap_measures_", Country_3[i])),

                                                          heights = c(10, 10),
                                                          #widths=c(2.3, 2.3, 2.3),
                                                          ncol = 1, nrow = 2,
                                                          align = "v"))
  }else{
    assign(paste0("total_plot_", Country_3[i]), ggarrange(ggplot()))
  }
```

```
  if(nrow(df_study_print) != 0){
    assign(paste0("total_plot_", Country_3[i],"_3"), ggarrange(get(paste0("study_table_", Country_3[i])),
                                                               text_annotation,
                                                               #heights = c(3.2, 0.8),
                                                               #widths=c(2.3, 2.3),
                                                               ncol = 1, nrow = 2))
    assign(paste0("total_plot_", Country_3[i],"_4"), ggarrange(get(paste0("study_table_", Country_3[i])),
                                                               text_annotation1,
                                                               #heights = c(3.2, 0.8),
                                                               #widths=c(2.3, 2.3),
                                                               ncol = 1, nrow = 2)
    )
  }else{
    assign(paste0("total_plot_", Country_3[i],"_3"), ggarrange(text_annotation,
                                                               #heights = c(3.2, 0.8),
                                                               #widths=c(2.3, 2.3),
                                                               ncol = 1, nrow = 1)
    )
    assign(paste0("total_plot_", Country_3[i],"_4"), ggarrange(text_annotation1,
                                                               #heights = c(3.2, 0.8),
                                                               #widths=c(2.3, 2.3),
                                                               ncol = 1, nrow = 1)
    )
  }


  assign(paste0("total_plot_", Country_3[i],"_2"), ggarrange(get(paste0("total_plot_", Country_3[i])) ,
                                                             get(paste0("total_plot_", Country_3[i], "_3")),
                                                             heights = c(12, 7),
                                                             #widths=c(2.3, 2.3),
                                                             ncol = 1, nrow = 2)
  )
  assign(paste0("total_plot_", Country_3[i],"_5"), ggarrange(get(paste0("total_plot_", Country_3[i])) ,
                                                             get(paste0("total_plot_", Country_3[i], "_4")),
                                                             heights = c(12, 7),
                                                             #widths=c(2.3, 2.3),
                                                             ncol = 1, nrow = 2)
  )

  pdf(paste0("plots_2",Country_3[i],".pdf"), paper="a4")
  print(get(paste0("total_plot_", Country_3[i],"_2")))
  dev.off()

  pdf(paste0("plots_2_heatcases_",Country_3[i],".pdf"), paper="a4")
  print(get(paste0("total_plot_", Country_3[i])))
  dev.off()

  pdf(paste0("plots_2_heatcasestab_",Country_3[i],".pdf"), paper="a4")
  print(get(paste0("total_plot_", Country_3[i],"_5")))
  dev.off()

  png(paste0("plots_2",Country_3[i],".png"))
  print(get(paste0("total_plot_", Country_3[i],"_2")))
  dev.off()

  png(paste0("plots_2_heatcases_",Country_3[i],".png"))
  print(get(paste0("total_plot_", Country_3[i])))
  dev.off()

  png(paste0("plots_2_heatcasestab_",Country_3[i],".png"))
  print(get(paste0("total_plot_", Country_3[i],"_5")))
  dev.off()
}
```
