# Supplementary material for: Intra-Household and Close-Contact SARS-CoV-2 Transmission Among Children – a Systematic Review
Source: Front Pediatr. 2021 Apr 9;9:613292. doi: 10.3389/fped.2021.613292 (PMC8062727; doi:10.3389/fped.2021.613292)
Supplement: Supplementary file 2 [file Table_1.docx]

Supplementary Material

**Supplementary Table 2:** Characteristics of studies discussing viral load and viral shedding of SARS-CoV-2

| First Author | Country | Study status; type | Timing of study | N(children);  N(adults) | Method | age range (years): child; adult |
| --- | --- | --- | --- | --- | --- | --- |
| Chau et al. (65) | Vietnam | Published, Corrected Proof | 10.03.2020 - 05.04.2020 | 30 participants | prospective study at a quarantine center for COVID-19 in Ho Chi Minh City collecting epidemiological data and laboratory testing | 16-17; 18-60 |
| Wolf et al. (75) | Germany | Online Report | 24.01.2020 - not stated | 3; 2 | characterization of the family cluster regarding transmission details, epidemiological data and focus of the clinical presentation of the three child cases | 7months - 5 years; not stated |
| Baggio et al. (86) | Switzerland | Published, Peer Reviewed | 29.03.2020 - 23.04.2020 | 53; 352 | analysis of RT-PCR threshold cycle data derived from specimens collected in the context of prospective cohort studies at a single center | 0-19; > 20 |
| L’Huillier et al. (87) | Switzerland | Research Letter | 25.01.2020 - 31.03.2020 | 23 children | cell culture of SARS-CoV-2 isolated from specimens of the upper respiratory tract of 23 children with COVID-19 | 1month - 15,9years; none |
| Han et al. (88) | South Korea | Research letter | 08.03.2020 - 28.04.2020 | 12; 0 | analysis of clinical specimens from SARS-CoV-2 positive children regardless of disease severity using RT-PCR. | 27days – 16years; none |
| Xiao et al. (89) | China | Published, Peer Reviewed | 01.02.2020 – 14.02.2020 | 73 infected patients | analysis of several specimens including serum, swabs, stool etc. from hospitalized SARS-CoV-2 patients using RT-PCR and Histologic staining. | 10months – < 18years; 18-78 |
| He et al. (90) | China | Brief Communi-cation | 21.01.2020 – 14.02.2020 | 94 infected patients | analysis of throat swabs from SARS-CoV-2 positive patients with cases ranging from asymptomatic to moderate symptomatic at admission using RT-PCR. | Not stated |
| Hua et al. (91) | China | Published, Peer Reviewed | 01.01.2020 - 24.02.2020 | 417; 632 | retrospective analysis of the electronic database of the Zhejian Province and records of the Chinese Center for Disease Control and Prevention | 3months-14; 15-96 |
| Ma et al. (92) | China | Short Communi-cation | January 2020 – February 2020 | 6; 2 | Analysis of stool specimens from SARS-CoV-2 positive patients in their recovery phase using RT-PCR. | 11monts – 9years; 33-39 |
| Jones et al. (93) | Germany | Preprint | January 2020 - May 2020 | 3303 with positive overall results | retrospective analysis of RT-PCR threshold cycle data from patients tested positive for SARS-CoV-2 | 0-19; 20-99 |
